# Rarity of monodominance in hyperdiverse Amazonian forests

**DOI:** 10.1038/s41598-019-50323-9

**Published:** 2019-09-25

**Authors:** Hans ter Steege, Terry W. Henkel, Nora Helal, Beatriz S. Marimon, Ben Hur Marimon-Junior, Andreas Huth, Jürgen Groeneveld, Daniel Sabatier, Luiz de Souza Coelho, Diogenes de Andrade Lima Filho, Rafael P. Salomão, Iêda Leão Amaral, Francisca Dionízia de Almeida Matos, Carolina V. Castilho, Oliver L. Phillips, Juan Ernesto Guevara, Marcelo de Jesus Veiga Carim, Dairon Cárdenas López, William E. Magnusson, Florian Wittmann, Mariana Victória Irume, Maria Pires Martins, José Renan da Silva Guimarães, Jean-François Molino, Olaf S. Bánki, Maria Teresa Fernandez Piedade, Nigel C. A. Pitman, Abel Monteagudo Mendoza, José Ferreira Ramos, Bruno Garcia Luize, Evlyn Márcia Moraes de Leão Novo, Percy Núñez Vargas, Thiago Sanna Freire Silva, Eduardo Martins Venticinque, Angelo Gilberto Manzatto, Neidiane Farias Costa Reis, John Terborgh, Katia Regina Casula, Euridice N. Honorio Coronado, Juan Carlos Montero, Ted R. Feldpausch, Alvaro Duque, Flávia R. C. Costa, Nicolás Castaño Arboleda, Jochen Schöngart, Timothy J. Killeen, Rodolfo Vasquez, Bonifacio Mostacedo, Layon O. Demarchi, Rafael L. Assis, Chris Baraloto, Julien Engel, Pascal Petronelli, Hernán Castellanos, Marcelo Brilhante de Medeiros, Adriano Quaresma, Marcelo Fragomeni Simon, Ana Andrade, José Luís Camargo, Susan G. W. Laurance, William F. Laurance, Lorena M. Rincón, Juliana Schietti, Thaiane R. Sousa, Emanuelle de Sousa Farias, Maria Aparecida Lopes, José Leonardo Lima Magalhães, Henrique Eduardo Mendonça Nascimento, Helder Lima de Queiroz, Gerardo A. Aymard C., Roel Brienen, Juan David Cardenas Revilla, Ima Célia Guimarães Vieira, Bruno Barçante Ladvocat Cintra, Pablo R. Stevenson, Yuri Oliveira Feitosa, Joost F. Duivenvoorden, Hugo F. Mogollón, Alejandro Araujo-Murakami, Leandro Valle Ferreira, José Rafael Lozada, James A. Comiskey, José Julio de Toledo, Gabriel Damasco, Nállarett Dávila, Freddie Draper, Roosevelt García-Villacorta, Aline Lopes, Alberto Vicentini, Alfonso Alonso, Francisco Dallmeier, Vitor H. F. Gomes, Jon Lloyd, David Neill, Daniel Praia Portela de Aguiar, Luzmila Arroyo, Fernanda Antunes Carvalho, Fernanda Coelho de Souza, Dário Dantas do Amaral, Kenneth J. Feeley, Rogerio Gribel, Marcelo Petratti Pansonato, Jos Barlow, Erika Berenguer, Joice Ferreira, Paul V. A. Fine, Marcelino Carneiro Guedes, Eliana M. Jimenez, Juan Carlos Licona, Maria Cristina Peñuela Mora, Boris Villa, Carlos Cerón, Paul Maas, Marcos Silveira, Juliana Stropp, Raquel Thomas, Tim R. Baker, Doug Daly, Kyle G. Dexter, Isau Huamantupa-Chuquimaco, William Milliken, Toby Pennington, Marcos Ríos Paredes, Alfredo Fuentes, Bente Klitgaard, José Luis Marcelo Pena, Carlos A. Peres, Miles R. Silman, J. Sebastián Tello, Jerome Chave, Fernando Cornejo Valverde, Anthony Di Fiore, Renato Richard Hilário, Juan Fernando Phillips, Gonzalo Rivas-Torres, Tinde R. van Andel, Patricio von Hildebrand, Janaína Costa Noronha, Edelcilio Marques Barbosa, Flávia Rodrigues Barbosa, Luiz Carlos de Matos Bonates, Rainiellen de Sá Carpanedo, Hilda Paulette Dávila Doza, Émile Fonty, Ricardo GómeZárate z, Therany Gonzales, George Pepe Gallardo Gonzales, Bruce Hoffman, André Braga Junqueira, Yadvinder Malhi, Ires Paula de Andrade Miranda, Linder Felipe Mozombite Pinto, Adriana Prieto, Domingos de Jesus Rodrigues, Agustín Rudas, Ademir R. Ruschel, Natalino Silva, César I. A. Vela, Vincent Antoine Vos, Egleé L. Zent, Stanford Zent, Bianca Weiss Albuquerque, Angela Cano, Yrma Andreina Carrero Márquez, Diego F. Correa, Janaina Barbosa Pedrosa Costa, Bernardo Monteiro Flores, David Galbraith, Milena Holmgren, Michelle Kalamandeen, Marcelo Trindade Nascimento, Alexandre A. Oliveira, Hirma Ramirez-Angulo, Maira Rocha, Veridiana Vizoni Scudeller, Rodrigo Sierra, Milton Tirado, Maria Natalia Umaña Medina, Geertje van der Heijden, Emilio Vilanova Torre, Corine Vriesendorp, Ophelia Wang, Kenneth R. Young, Manuel Augusto Ahuite Reategui, Cláudia Baider, Henrik Balslev, Sasha Cárdenas, Luisa Fernanda Casas, William Farfan-Rios, Cid Ferreira, Reynaldo Linares-Palomino, Casimiro Mendoza, Italo Mesones, Armando Torres-Lezama, Ligia Estela Urrego Giraldo, Daniel Villarroel, Roderick Zagt, Miguel N. Alexiades, Edmar Almeida de Oliveira, Karina Garcia-Cabrera, Lionel Hernandez, Walter Palacios Cuenca, Susamar Pansini, Daniela Pauletto, Freddy Ramirez Arevalo, Adeilza Felipe Sampaio, Elvis H. Valderrama Sandoval, Luis Valenzuela Gamarra, Aurora Levesley, Georgia Pickavance, Karina Melgaço

**Affiliations:** 1 Biodiversity Dynamics, Naturalis Biodiversity Center, PO Box 9517, Leiden, 2300 RA The Netherlands; 2 Systems Ecology, Free University, De Boelelaan 1087, Amsterdam, 1081 HV The Netherlands; 3 Department of Biological Sciences, Humboldt State University, 1 Harpst Street, Arcata, CA 95521 USA; 4 Programa de Pós-Graduação em Ecologia e Conservação, Universidade do Estado de Mato Grosso, Nova Xavantina, MT Brazil; 5 Department of Ecological Modelling, Helmholtz Centre for Environmental Research - UFZ, Permoserstr. 15, Leipzig, 4318 Germany; 6 Institute of Forest Growth and Computer Sciences, Technische Universitaet Dresden, Postfach 1117, Tharandt, 1735 Germany; 7 AMAP, IRD, CIRAD, CNRS, INRA, Université de Montpellier, TA A-51/PS2, Bd. de la Lironde, A comprehensive, Montpellier, F-34398 France; 8 Coordenação de Biodiversidade, Instituto Nacional de Pesquisas da Amazônia - INPA, Av. André Araújo, 2936, Petrópolis, Manaus, AM 69067-375 Brazil; 9 Programa Professor Visitante Nacional Sênior na Amazônia - CAPES, Universidade Federal Rural da Amazônia, Av. Perimetral, s/n, Belém, PA Brazil; 10 Coordenação de Botânica, Museu Paraense Emílio Goeldi, Av. Magalhães Barata 376, C.P. 399, Belém, PA 66040-170 Brazil; 11 EMBRAPA – Centro de Pesquisa Agroflorestal de Roraima, BR 174, km 8 – Distrito Industrial, Boa Vista, RR 69301-970 Brazil; 12 School of Geography, University of Leeds, Woodhouse Lane, Leeds, LS2 9JT UK; 13 Grupo de Investigación en Biodiversidad, Medio Ambiente y Salud-BIOMAS, Universidad de las Américas, Campus Queri, Quito, Ecuador; 14 Keller Science Action Center, The Field Museum, 1400S. Lake Shore Drive, Chicago, IL 60605-2496 USA; 15 Departamento de Botânica, Instituto de Pesquisas Científicas e Tecnológicas do Amapá - IEPA, Rodovia JK, Km 10, Campus do IEPA da Fazendinha, Amapá, 68901-025 Brazil; 16 Herbario Amazónico Colombiano, Instituto SINCHI, Calle 20 No 5-44, Bogotá, DC Colombia; 17 Coordenação de Pesquisas em Ecologia, Instituto Nacional de Pesquisas da Amazônia - INPA, Av. André Araújo, 2936, Petrópolis, Manaus, AM 69067-375 Brazil; 18 Dep. of Wetland Ecology, Institute of Geography and Geoecology, Karlsruhe Institute of Technology - KIT, Josefstr.1, Rastatt, D-76437 Germany; 19 Biogeochemistry, Max Planck Institute for Chemistry, Hahn-Meitner Weg 1, Mainz, 55128 Germany; 20 Naturalis Biodiversity Center, PO Box 9517, Leiden, 2300 RA The Netherlands; 21 Coordenação de Dinâmica Ambiental, Instituto Nacional de Pesquisas da Amazônia - INPA, Av. André Araújo, 2936, Petrópolis, Manaus, AM 69067-375 Brazil; 22 Science and Education, The Field Museum, 1400S. Lake Shore Drive, Chicago, IL 60605-2496 USA; 23 Jardín Botánico de Missouri, Oxapampa, Pasco Peru; 24 Departamento de Ecologia, Universidade Estadual Paulista - UNESP – Instituto de Biociências – IB, Av. 24A, 1515, Bela Vista, Rio Claro, SP 13506-900 Brazil; 25 Divisao de Sensoriamento Remoto – DSR, Instituto Nacional de Pesquisas Espaciais – INPE, Av. dos Astronautas, 1758, Jardim da Granja, São José dos Campos, SP 12227-010 Brazil; 26 Herbario Vargas, Universidad Nacional de San Antonio Abad del Cusco, Avenida de la Cultura, Nro 733, Cusco, Cuzco Peru; 27 Departamento de Geografia, Universidade Estadual Paulista -UNESP – Instituto de Geociências e Ciências Extas – IGCE, Bela Vista, Rio Claro, SP 13506-900 Brazil; 28 Centro de Biociências, Departamento de Ecologia, Universidade Federal do Rio Grande do Norte, Av. Senador Salgado Filho, 3000, Natal, RN 59072-970 Brazil; 29 Departamento de Biologia, Universidade Federal de Rondônia, Rodovia BR 364s/n Km 9,5 - Sentido Acre, Unir, Porto Velho, RO 76.824-027 Brazil; 30 Programa de Pós- Graduação em Biodiversidade e Biotecnologia PPG- Bionorte, Universidade Federal de Rondônia, Campus Porto Velho Km 9,5 bairro Rural, Porto Velho, RO 76.824-027 Brazil; 31 Department of Biology and Florida Museum of Natural History, University of Florida, Gainesville, FL 32611 USA; 32 Centre for Tropical Environmental and Sustainability Science and College of Science and Engineering, James Cook University, Cairns, Queensland 4870 Australia; 33 Instituto de Investigaciones de la Amazonía Peruana (IIAP), Av. A. Quiñones km 2,5, Iquitos, Loreto 784 Peru; 34 Instituto Boliviano de Investigacion Forestal, Av. 6 de agosto #28, Km. 14, Doble via La Guardia, Casilla 6204, Santa Cruz, Santa Cruz Bolivia; 35 Geography, College of Life and Environmental Sciences, University of Exeter, Rennes Drive, Exeter, EX4 4RJ UK; 36 Departamento de Ciencias Forestales, Universidad Nacional de Colombia, Calle 64 x Cra 65, Medellín, Antioquia 1027 Colombia; 37 Agteca-Amazonica, Santa Cruz, Bolivia; 38 Facultad de Ciencias Agrícolas, Universidad Autónoma Gabriel René Moreno, Santa Cruz, Santa Cruz Bolivia; 39 Natural History Museum, University of Oslo, Postboks 1172, Oslo, 318 Norway; 40 International Center for Tropical Botany (ICTB) Department of Biological Sciences, Florida International University, 11200 SW 8th Street, OE 243, Miami, FL 33199 USA; 41 Cirad UMR Ecofog, AgrosParisTech, CNRS, INRA, Univ Guyane, Campus agronomique, Kourou Cedex, 97379 France; 42 Centro de Investigaciones Ecológicas de Guayana, Universidad Nacional Experimental de Guayana, Calle Chile, urbaniz Chilemex, Puerto Ordaz, Bolivar Venezuela; 43 Prédio da Botânica e Ecologia, Embrapa Recursos Genéticos e Biotecnologia, Parque Estação Biológica, Av. W5 Norte, Brasilia, DF 70770-917 Brazil; 44 Projeto Dinâmica Biológica de Fragmentos Florestais, Instituto Nacional de Pesquisas da Amazônia - INPA, Av. André Araújo, 2936, Petrópolis, Manaus, AM 69067-375 Brazil; 45 Laboratório de Ecologia de Doenças Transmissíveis da Amazônia (EDTA), Instituto Leônidas e Maria Deane, Fiocruz, Rua Terezina, 476, Adrianópolis, Manaus, AM 69060-001 Brazil; 46 Programa de Pós-graduação em Biodiversidade e Saúde, Instituto Oswaldo Cruz - IOC/FIOCRUZ, Pav. Arthur Neiva – Térreo, Av. Brasil, 4365 – Manguinhos, Rio de Janeiro, RJ 21040-360 Brazil; 47 Instituto de Ciências Biológicas, Universidade Federal do Pará, Av. Augusto Corrêa 01, Belém, PA 66075-110 Brazil; 48 Programa de Pós-Graduação em Ecologia, Universidade Federal do Pará, Av. Augusto Corrêa 01, Belém, PA 66075-110 Brazil; 49 Embrapa Amazônia Oriental, Trav. Dr. Enéas Pinheiro s/no., Belém, PA 66095-100 Brazil; 50 Diretoria Técnico-Científica, Instituto de Desenvolvimento Sustentável Mamirauá, Estrada do Bexiga, 2584, Tefé, AM 69470-000 Brazil; 51 Programa de Ciencias del Agro y el Mar, Herbario Universitario (PORT), UNELLEZ-Guanare, Guanare, Portuguesa 3350 Venezuela; 52 Laboratorio de Ecología de Bosques Tropicales y Primatología, Universidad de los Andes, Carrera 1 # 18a- 10, Bogotá, DC 111711 Colombia; 53 Programa de Pós-Graduação em Biologia (Botânica), Instituto Nacional de Pesquisas da Amazônia - INPA, Av. André Araújo, 2936, Petrópolis, Manaus, AM 69067-375 Brazil; 54 Institute of Biodiversity and Ecosystem Dynamics, University of Amsterdam, Sciencepark 904, Amsterdam, 1098 XH The Netherlands; 55 Endangered Species Coalition, 8530 Geren Rd., Silver Spring, MD 20901 USA; 56 Museo de Historia Natural Noel Kempff Mercado, Universidad Autónoma Gabriel Rene Moreno, Avenida Irala 565 Casilla Post al 2489, Santa Cruz, Santa Cruz Bolivia; 57 Facultad de Ciencias Forestales y Ambientales, Instituto de Investigaciones para el Desarrollo Forestal, Universidad de los Andes, Via Chorros de Milla, 5101 Mérida, Mérida Venezuela; 58 Inventory and Monitoring Program, National Park Service, 120 Chatham Lane, Fredericksburg, VA 22405 USA; 59 Center for Conservation and Sustainability, Smithsonian Conservation Biology Institute, 1100 Jefferson Dr. SW, Suite 3123, Washington, DC 20560-0705 USA; 60 Universidade Federal do Amapá, Ciências Ambientais, Rod. Juscelino Kubitschek km2, Macapá, AP 68902-280 Brazil; 61 Department of Integrative Biology, University of California, Berkeley, CA 94720-3140 USA; 62 Biologia Vegetal, Universidade Estadual de Campinas, Caixa Postal 6109, Campinas, SP 13.083-970 Brazil; 63 Department of Global Ecology, Carnegie Institution for Science, 260 Panama St., Stanford, CA 94305 USA; 64 Institute of Molecular Plant Sciences, University of Edinburgh, Mayfield Rd, Edinburgh, EH3 5LR UK; 65 Tropical Diversity Section, Royal Botanic Garden Edinburgh, 20a Inverleith Row, Edinburgh, Scotland EH3 5LR UK; 66 Department of Ecology, University of Brasilia, Brasilia, DF 70904-970 Brazil; 67 Programa de Pós-Graduação em Ciência Ambientais, Universidade Federal do Pará, Rua Augusto Corrêa 01, Belém, PA 66075-110 Brazil; 68 Faculty of Natural Sciences, Department of Life Sciences, Imperial College London, Silwood Park, South Kensington Campus, London, SW7 2AZ UK; 69 Ecosistemas, Biodiversidad y Conservación de Especies, Universidad Estatal Amazónica, Km. 2 1/2 vía a Tena (Paso Lateral), Puyo, Pastaza Ecuador; 70 Universidade Federal de Minas Gerais, Instituto de Ciências Biológicas, Departamento de Genética, Ecologia e Evolução, Av. Antônio Carlos, 6627 Pampulha, Belo Horizonte, MG 31270-901 Brazil; 71 Department of Biology, University of Miami, Coral Gables, FL 33146 USA; 72 Fairchild Tropical Botanic Garden, Coral Gables, FL 33156 USA; 73 Diretoria de Pesquisas Científicas, Instituto de Pesquisas Jardim Botânico do Rio de Janeiro, Rio de Janeiro, RJ Brazil; 74 Instituto de Biociências - Dept. Ecologia, Universidade de Sao Paulo - USP, Rua do Matão, Trav. 14, no. 321, Cidade Universitária, São Paulo, SP 05508-090 Brazil; 75 Lancaster Environment Centre, Lancaster University, Lancaster, Lancashire LA1 4YQ UK; 76 Environmental Change Institute, University of Oxford, Oxford, Oxfordshire OX1 3QY UK; 77 Empresa Brasileira de Pesquisa Agropecuária, Embrapa Amapá, Rod. Juscelino Kubitschek km 5, Macapá, Amapá 68903-419 Brazil; 78 Grupo de Investigación en Tecnologías de la Información y Medio Ambiente, Instituto Tecnológico de Antioquia - Institución Universitaria, Calle 78B No. 72A-220, Medellín, Colombia; 79 Universidad Regional Amazónica IKIAM, Km 7 via Muyuna, Tena, Napo Ecuador; 80 Escuela de Biología Herbario Alfredo Paredes, Universidad Central, Ap. Postal 17.01.2177, Quito, Pichincha Ecuador; 81 Taxonomy and Systematics, Naturalis Biodiversity Center, PO Box 9517, Leiden, 2300 RA The Netherlands; 82 Museu Universitário/Centro de Ciências Biológicas e da Natureza/Laboratório de Botânica e Ecologia Vegetal, Universidade Federal do Acre, Rio Branco, AC 69915-559 Brazil; 83 Institute of Biological and Health Sciences, Federal University of Alagoas, Av. Lourival Melo Mota, s/n, Tabuleiro do Martins, Maceio, AL 57072-970 Brazil; 84 Iwokrama International Centre for Rainforest Conservation, Georgetown, Guyana; 85 New York Botanical Garden, 2900 Southern Blvd, Bronx, New York, NY 10458-5126 USA; 86 School of Geosciences, University of Edinburgh, 201 Crew Building, King’s Buildings, Edinburgh, EH9 3JN UK; 87 Natural Capital and Plant Health, Royal Botanic Gardens, Kew, Richmond, Surrey TW9 3AB UK; 88 Servicios de Biodiversidad EIRL, Jr. Independencia 405, Iquitos, Loreto 784 Peru; 89 Herbario Nacional de Bolivia, Universitario UMSA, Casilla 10077 Correo Central, La Paz, La Paz Bolivia; 90 Center for Conservation and Sustainable Development, Missouri Botanical Garden, P.O. Box 299, St. Louis, MO 63166-0299 USA; 91 Department for Identification & Naming, Royal Botanic Gardens, Kew, Richmond, Surrey TW9 3AB UK; 92 Department of Forestry Management, Universidad Nacional Agraria La Molina, Avenido La Molina, Apdo. 456, La Molina, Lima, Peru; 93 School of Environmental Sciences, University of East Anglia, Norwich, NR4 7TJ UK; 94 Biology Department and Center for Energy, Environment and Sustainability, Wake Forest University, 1834 Wake Forest Rd, Winston Salem, NC 27106 USA; 95 Laboratoire Evolution et Diversité Biologique, CNRS and Université Paul Sabatier, UMR 5174 EDB, Toulouse, 31000 France; 96 Andes to Amazon Biodiversity Program, Madre de Dios, Madre de Dios Peru; 97 Department of Anthropology, University of Texas at Austin, SAC 5.150, 2201 Speedway Stop C3200, Austin, TX 78712 USA; 98 Fundación Puerto Rastrojo, Cra 10 No. 24-76 Oficina 1201, Bogotá, DC Colombia; 99 Colegio de Ciencias Biológicas y Ambientales-COCIBA & Galapagos Institute for the Arts and Sciences-GAIAS, Universidad San Francisco de Quito-USFQ, Quito, Pichincha Ecuador; 100 Department of Wildlife Ecology and Conservation, University of Florida, 110 Newins-Ziegler Hall, Gainesville, FL 32611 USA; 101 Fundación Estación de Biología, Cra 10 No. 24-76 Oficina 1201, Bogotá, DC Colombia; 102 ICNHS, Federal University of Mato Grosso, Av. Alexandre Ferronato 1200, Setor Industrial, Sinop, MT 78.557-267 Brazil; 103 Direction régionale de la Guyane, ONF, Cayenne, F-97300 French Guiana; 104 PROTERRA, Instituto de Investigaciones de la Amazonía Peruana (IIAP), Av. A. Quiñones km 2,5, Iquitos, Loreto 784 Peru; 105 ACEER Foundation, Jirón Cusco No. 370, Puerto Maldonado, Madre de Dios Peru; 106 Amazon Conservation Team, Doekhieweg Oost #24, Paramaribo, Suriname; 107 Institut de Ciència i Tecnologia Ambientals, Universitat Autònoma de Barcelona, 08193 Bellaterra, Barcelona Spain; 108 Environmental Change Institute, Oxford University Centre for the Environment, Dyson Perrins Building, South Parks Road, Oxford, England OX1 3QY UK; 109 Instituto de Ciencias Naturales, Universidad Nacional de Colombia, Apartado 7945, Bogotá, DC Colombia; 110 Instituto de Ciência Agrárias, Universidade Federal Rural da Amazônia, Av. Presidente Tancredo Neves 2501, Belém, PA 66.077-830 Brazil; 111 Escuela Profesional de Ingeniería Forestal, Universidad Nacional de San Antonio Abad del Cusco, Jirón San Martín 451, Puerto Maldonado, Madre de Dios Peru; 112 Universidad Autónoma del Beni José Ballivián, Campus Universitario Final, Av. Ejercito, Riberalta, Beni Bolivia; 113 Laboratory of Human Ecology, Instituto Venezolano de Investigaciones Científicas - IVIC, Ado 20632, Caracas, Caracas 1020 A Venezuela; 114 Cambridge University Botanic Garden, 1 Brookside., Cambridge, CB2 1JE UK; 115 School of Agriculture and Food Sciences - ARC Centre of Excellence for Environmental Decisions CEED, The University of Queensland, St. Lucia, QLD 4072 Australia; 116 University of Campinas, Plant Biology Department, Rua Monteiro Lobato, 255, Cidade Universitária Zeferino Vaz, Barão Geraldo, Campinas, São Paulo, CEP 13083-862 Brazil; 117 Resource Ecology Group, Wageningen University & Research, Droevendaalsesteeg 3a, Lumen, building number 100, Wageningen, Gelderland 6708 PB The Netherlands; 118 Laboratório de Ciências Ambientais, Universidade Estadual do Norte Fluminense, Av. Alberto Lamego 2000, Campos dos Goyatacazes, RJ 28013-620 Brazil; 119 Instituto de Investigaciones para el Desarrollo Forestal (INDEFOR), Universidad de los Andes, Conjunto Forestal, 5101 Mérida, Mérida Venezuela; 120 Departamento de Biologia, Universidade Federal do Amazonas - UFAM – Instituto de Ciências Biológicas – ICB1, Av General Rodrigo Octavio 6200, Manaus, AM 69080-900 Brazil; 121 GeoIS, El Día 369y El Telégrafo, 3° Piso, Quito, Pichincha Ecuador; 122 Department of Biology, University of Maryland, College Park, MD 20742 USA; 123 University of Nottingham, University Park, Nottingham, NG7 2RD UK; 124 School of Environmental and Forest Sciences, University of Washington, Seattle, WA 98195-2100 USA; 125 Environmental Science and Policy, Northern Arizona University, Flagstaff, AZ 86011 USA; 126 Geography and the Environment, University of Texas at Austin, 305 E. 23rd Street, CLA building, Austin, TX 78712 USA; 127 Medio Ambiente, PLUSPRETOL, Iquitos, Loreto Peru; 128 The Mauritius Herbarium, Agricultural Services, Ministry of Agro-Industry and Food Security, Reduit, 80835 Mauritius; 129 Department of Bioscience, Aarhus University, Building 1540 Ny Munkegade, Aarhus C, Aarhus, DK-8000 Denmark; 130 FOMABO, Manejo Forestal en las Tierras Tropicales de Bolivia, Sacta, Cochabamba Bolivia; 131 Escuela de Ciencias Forestales (ESFOR), Universidad Mayor de San Simon (UMSS), Sacta, Cochabamba Bolivia; 132 Tropenbos International, Lawickse Allee 11 PO Box 232, Wageningen, 6700 AE The Netherlands; 133 School of Anthropology and Conservation, University of Kent, Marlowe Building, Canterbury, Kent CT2 7NR UK; 134 Herbario Nacional del Ecuador, Universidad Técnica del Norte, Quito, Pichincha Ecuador; 135 Instituto de Biodiversidade e Floresta, Universidade Federal do Oeste do Pará, Rua Vera Paz, Campus Tapajós, Santarém, PA 68015-110 Brazil; 136 Facultad de Biologia, Universidad Nacional de la Amazonia Peruana, Pevas 5ta cdra, Iquitos, Loreto Peru; 137 Department of Biology, University of Missouri, St. Louis, MO 63121 USA

**Keywords:** Biodiversity, Forest ecology

## Abstract

Tropical forests are known for their high diversity. Yet, forest patches do occur in the tropics where a single tree species is dominant. Such “monodominant” forests are known from all of the main tropical regions. For Amazonia, we sampled the occurrence of monodominance in a massive, basin-wide database of forest-inventory plots from the Amazon Tree Diversity Network (ATDN). Utilizing a simple defining metric of at least half of the trees ≥ 10 cm diameter belonging to one species, we found only a few occurrences of monodominance in Amazonia, and the phenomenon was not significantly linked to previously hypothesized life history traits such wood density, seed mass, ectomycorrhizal associations, or *Rhizobium* nodulation. In our analysis, coppicing (the formation of sprouts at the base of the tree or on roots) was the only trait significantly linked to monodominance. While at specific locales coppicing or ectomycorrhizal associations may confer a considerable advantage to a tree species and lead to its monodominance, very few species have these traits. Mining of the ATDN dataset suggests that monodominance is quite rare in Amazonia, and may be linked primarily to edaphic factors.

## Introduction

Tropical forests contain Earth’s highest levels of biodiversity. Over 250 tree species ≥10 cm diameter can be found in a 1-ha plot of the continental lowland tropics, whereas a similar area in the most diverse temperate broadleaf forest may hold 20–30 species^1,2^. Within such hyperdiverse tropical forests, however, patches occur that are dominated by a single tree species (hereafter “monodominant”). The earliest reports of tropical monodominant forests in the Amazon Basin were given by the explorers Spruce and Wallace^3–5^, who noted forests highly dominated by *Eperua purpurea* Benth. and *E. leucantha* Benth. on the white sands of the Upper Rio Negro Basin. Later Hamilton-Rice^6^ discovered large stands dominated by *Peltogyne gracilipes* Ducke (Fabaceae) during a 1924–25 expedition to northern Brazil. These perplexing single-dominant forests did not fit the traditional perception of uniformly tree-diverse tropical forests^6^. Similarly, the botanical explorer J. G. Myers, on his trek through the Pakaraima Mountains of then British Guiana, observed forests heavily dominated by either *Peltogyne* sp., *Micrandra glabra* Schultes (Euphorbiaceae), or multi-stemmed *Dicymbe corymbosa* Spruce ex. Benth. (Fabaceae)^7^. Monodominance has since been documented in all the tropical regions^8–13^. A stand has traditionally been considered monodominant when the number of canopy-level trees belonging to the same species is ≥60%^9,14^. Monodominant forests are “persistently dominant” when the dominant species dominates all strata/age classes in the stand, and will remain monodominant through time (i.e. late-successional, and not being a dominant, but transient, earlier successional stage).

Several mechanisms have been suggested to explain monodominance but a full understanding has yet to be achieved. Monodominance remains a topic of intensive research with controversial findings e.g.^15,16^. The term ‘classical monodominance’ was introduced by Peh^17^ and is defined as the occurrence of monodominant forests with environmental conditions similar to those of adjacent mixed-forests. Several studies, however, have revealed environmental differences between these forests, previously undetected. For example, soil nutrient or moisture availability may vary between monodominant and mixed forests^10,11,13,18–21^. Conversely, other studies have indicated that soil characteristics cannot alone explain monodominance^13,22–26^.

Peh *et al*.^15^ summarized several of these contrasting studies on different continents and constructed a conceptual mechanistic framework that could explain monodominance in tropical forests. They suggested that monodominance is likely to emerge under a combination of mechanisms. Furthermore, the combination of traits and mechanisms leading to monodominance can differ between tree species and tropical areas^11,16,21,24^. Peh *et al*.^15^ hypothesized potential pathways to monodominance as based on two well-studied monodominant systems. The first pathway was based on the Afro-tropical, ectomycorrhizal (EM), monodominant canopy tree *Gilbertiodendron dewevrei* (De Wild.) J. Léonard. The most important mechanisms described for this species were based on seedling shade-tolerance and slow decomposition of leaves, resulting in deep leaf litter. Slow decomposition and deep leaf litter affect soil nutrient cycling and could negatively influence the survival of individuals of many species. These conditions could be advantageous for large seeded trees because they have more reserves for germination^11^. Trees with large seeds also tend to have shade-tolerant seedlings; seedlings of *G. dewevrei* are well adapted to the heavily shaded understory, resulting in a competitive advantage over non-shade tolerant pioneer species^11,14^. While not fully considered by Peh, *et al*.^15^, it is well-established that *G. dewevrei* adults are heavily EM throughout their trans-Congo range and that seedlings of the species share many EM fungal symbionts with their parents^27^. *Gilbertiodendron dewevrei*, under a minimal disturbance regime, could attain monodominance, as is described by the mechanisms of Peh, *et al*.^15^. This pathway was further examined by Kazmierczak, *et al*.^16^, who constructed a model demonstrating that species can obtain monodominance by possessing the intrinsic traits of seeds with large mass and low dispersibility.

The second example pathway was based on *Dicymbe corymbosa*, a Neotropical species. This species is a mast fruiting tree, which shows coppicing (the formation of sprouts at the base of the tree or on the roots) of shoots and roots^28^. It has been suggested that there is a link between mast seeding and EM associations that would lead to satiation of seed predators and increased seedling densities^29–31^. Henkel^23^ indicated that EM associations might also promote coppicing of shoots and roots by enhancing host plant nutrient supplies. Peh *et al*. pointed out that such positive feedbacks could, over time, result in the dominance of a tree species via competitive exclusion^15^. While documented examples of monodominant forests exist for the South American tropics, almost all cases are currently known from the Guiana Shield of the region’s northeast. Given the immensity of greater Amazonia, it begs the questions of how widespread monodominant forests might be, what tree species are involved, where they occur, and what environmental drivers are involved.

Here we examine the occurrence of monodominance within the context of a large plot network in Amazonia, the single largest, and arguably the richest, tropical forest on earth (Fig. S1). In line with the earlier concept of hyperdominance^32^, which was defined by the most common species that make up half of all trees across a region, here we call a site monodominant when a single species of tree constitutes more than half of the individual trees ≥10 cm diameter at breast height (dbh) in a stand of ~1 ha. Questions addressed were: (1) How common or rare is monodominance in Amazonia? (2) Which tree species can become monodominant? (3) Does monodominance occur more frequently in certain families? (4) Which traits characterize monodominant species? (5) In which regions do monodominant trees occur? and, 6) What environmental factors may drive monodominance? Given the paucity of published records from Amazonia, we tested not only for monodominance but also for lower dominance levels. As possible causes of monodominance, we investigated four main mechanisms:

1. *Competitive exclusion*. Monodominant forest in the tropics may develop when the forest does not experience large-scale disturbance over a long time period^9^. This mechanism is based on a study of Eggeling^33^, who compared tropical rainforest in Uganda in different successional stages. Eggeling showed that over the years, when no disturbance occurred, colonizing stands developed into climax stands with low species diversity, dominated by a few shade-tolerant species. This study led to the development of the intermediate-disturbance hypothesis (IDH)^34^. The IDH posits that a lack of (internal or external) disturbance leads to unconstrained succession and finally competitive exclusion, where the species that is best adapted to the environmental conditions will out-compete all other species and attain dominance^34,35^. Under such a mechanism, lowland tropical rainforests typically would not reach this endpoint due to frequent but spatiotemporally stochastic canopy-disturbing tree falls that allow influx of early-successional species into local gap areas, overall promoting persistence of high tree alpha-diversity. The IDH has been supported by evidence in some tropical rain forests^36,37^ but may have little effect on actual tree diversity^38^. If a lack of disturbance leads to competitive exclusion, we expect highest dominance in mature forests with the lowest numbers of pioneer species.
2. *Traits linked to above ground competition for light and space*. Functional traits may indicate a species-specific ecological strategy. For example, seed size and wood density give an indication of a species’ mode of establishment, growth rate, and survival, and have been used to characterize pioneer versus climax species^37,39^. If multiple functional traits of a monodominant species differ from those of non-dominant species, they could indicate a distinctive strategy leading to monodominance. However, if the functional traits differ *between* monodominant species, this would suggest that different mechanisms drive the trajectory to dominance^9^. Other hypotheses for monodominance are associated with specific competitive traits, such as seed size, where large seeds have low dispersal ability and seedlings establish near parent trees, leading to conspecific replacement over time^11,16^. Furthermore, the deep litter layers that have been found in monodominant forests could act as a physical barrier for seedling establishment, where large seeds have an advantage over small-seeded species, as they have ample reserves to germinate and establish root systems^11^. However, small-seeded monodominant species have also been documented, and studies have shown that deep leaf litter does not always affect seedling establishment of non-dominant species. This suggests that large seeds could be a contributing, but not the sole, trait for monodominance^15,40^. The formation of coppices has been linked to monodominance^23,28,41,42^. Coppicing involves the formation of multiple shoots at the base of the tree’s stem or from the root system in the absence of major crown injury. The phenomenon allows an individual to persist indefinitely in one location, as one or more shoots may take over when the original stem dies. However, coppicing has so far been found only in a handful of species of a few Amazonian tree genera. If competitive traits lead to monodominance we expect highest dominance by species with traits that are linked with competitive ability.
3. *Competitive traits linked to root-soil interactions*. A prominent hypothesis for how monodominance can emerge involves EM symbiosis creating a nutritional advantage for an EM-monodominant tree species with regard to establishment and survival^9,43–46^. The EM association consists of a mutualistic symbiosis between plant roots and fungi in which soil nutrients are provided by the fungus to the plant. It is striking that although most tropical trees are arbuscular mycorrhizal (AM), many monodominant tree species have EM associations^47,48^. The mechanisms behind this relationship are still not fully understood, but most likely involve plant-soil feedback mechanisms in which the local soil-litter conditions are altered in ways favouring the dominant EM tree species^9,18,44,49^. While both EM and AM fungi are dependent on their host plant for carbohydrate nutrition, and must obtain soil mineral nutrients for transfer to the plant, EM fungi have the enzymatic capacity to access organic forms of mineral nutrients directly from litter while avoiding major cellulolysis^50^. In systems dominated by EM plants, this mechanism would leave little for AM fungi, which are dependent on mineralized forms of nutrients for uptake. This implies direct competition between these fungi for mineral nutrients^50,51^. As a result, EM fungi may lead to slower overall decomposition (by mining of organic minerals and thus reducing the rate of saprotrophic cellulolysis) and reduced mineral nutrient availability for AM trees, this giving EM trees a competitive advantage^9,52^.
  Reliance on the “EM mechanism” to explain tropical monodominance is, however, fraught with difficulties, as (1) EM is not exclusively found in monodominant species; (2) monodominant species also occur without EM; (3) some monodominant species possess a combination of EM and AM; and (4) EM may not necessarily slow decomposition rates in tropical forests^18,44,53,54^. Therefore, we also tested other root-soil interactions including nitrogen (N-) fixation and aluminium accumulation that have yet to be linked to monodominance but could confer competitive advantages on nutrient poor or toxic soils. In the tropics, N-fixation occurs primarily in Fabaceae. While fixation leads to higher nitrogen in leaves of N-fixing species, especially in the wet tropics^55^, N-fixing Fabaceae do not dominate the most oligotrophic Amazonian ecosystems^56^, instead appear to have the greatest advantage in tropical dry successional forests^57^. Aluminium accumulation is found predominantly in a select number of families (e.g. Rubiaceae, Melastomataceae, Myrtaceae and Vochysiaceae [for Amazonian families])^58–61^, including a relation with monodominance (*Vochysia divergens*) in wet areas in the Brazilian Pantanal^62^. Large numbers of non-monodominant aluminium accumulators are found in the dry Cerrado areas e.g.^61^. If root-soil interactions are important drivers of competition, we expect EM, nodulating or aluminium accumulating species to be monodominant more frequently than expected by chance.
4. *Area*. All Amazon soil types present one or more stress factors to trees. For example, white sand soils are often dry and always low in nutrients, *igapó* and *várzea* both experience a single pulse of short to long-term flooding (up to 300 days), *igapó* soils are nutrient poor, *várzea* soils are nutrient rich, swamp soils are nearly permanently flooded or waterlogged with low oxygen tension, and *terra firme* soils are high in potentially toxic iron and aluminium. Because of several trade-offs, a tree species cannot be a good competitor on all of these soils e.g.^63,64^. Consequently, most common Amazonian tree species have a demonstrable preference for one of these soil types^32^. These tests often fail for rare species, either because they are non-preferential or are too infrequent to allow for a quantitative test^32^. With an assumption that all species in Amazonia have a near perfect habitat preference, we should expect that the total area of the soil types (and their level of fragmentation) has an effect on their tree species richness, with larger areas having more species^65–67^. Thus, a fourth possible mechanism for monodominance could be related to species-area relationships, where area is a controlling factor for species richness and dominance^66,68^. If the area of a distinct ‘edaphic forest type’ controls species richness, we expect monodominance to be more often found in plots in forests types that are small in total areal extent.

Because domestication has previously been linked to dominance in Amazonia^69^, we also investigated whether domesticated species are linked to monodominance.

## Results

**Figure 1:**
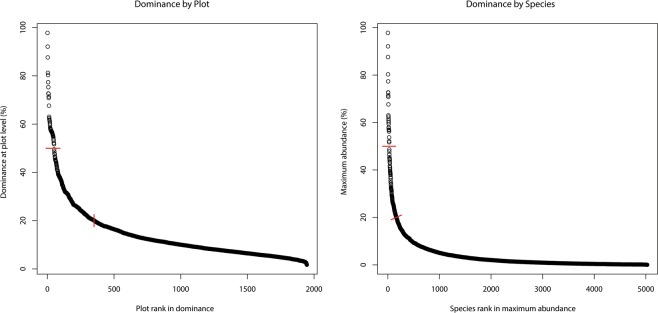
(**A)** Dominance at plot level (= relative abundance of the most abundant species of each plot) of 1946 inventory plots in Amazonia. Plots are ranked in order from high to low dominance. (**B**) Maximum relative abundance for each species (5029 species) found in 1946 inventory plots in Amazonia. Species are ranked from high to low maximum abundance. In each graph the red lines indicate 20 and 50% dominance.

Only 50 plots (2.6% of all plots) had levels of dominance over 50% of individuals >10 cm dbh of a single tree species (Fig. 1A) - for classical monodominance [>60%] these numbers were 19 plots (0.98%). In fact only 350 plots (18% of all plots) had dominance levels over 20%. Only 26 species (0.50% of all species) attained levels of dominance of ≥50%: *Eschweilera tenuifolia, Micrandra glabra, Ruizterania retusa, Pachira nitida, Machaerium hirtum, Spirotropis longifolia, Tabebuia aurea, Mauritia flexuosa, Brosimum rubescens, Lueheopsis hoehnei, Micrandra sprucei, Dicymbe corymbosa, Eperua falcata, Triplaris weigeltiana, Phyllanthus elsiae, Digomphia densicoma, Mora excelsa, Vitex cymosa, Euterpe oleracea, Oxandra polyantha, Macrolobium multijugum, Tachigali vaupesiana, Pachira brevipes, Astrocaryum macrocalyx, Attalea speciosa, Astrocaryum murumuru* (for species authorities see ter Steege, *et al*.^70^). The great majority of species (4863, 97%) did not attain 20% dominance or more. Stand-level dominant species, thus, account for a tiny minority of the tree species in Amazonia (Fig. 1B). Data by species and plot are given in Appendix S1.

Twelve out of 117 tree families had species which showed monodominance: Annonaceae, Arecaceae, Bignoniaceae, Euphorbiaceae, Fabaceae, Lamiaceae, Lecythidaceae, Malvaceae, Moraceae, Phyllanthaceae, Polygonaceae, Vochysiaceae (Appendix S2). Although Fabaceae species are a very prominent component of Amazonian forests^32^, the family had only seven monodominant species in the plots (*Machaerium hirtum, Spirotropis longifolia, Eperua falcata, Dicymbe corymbosa, Mora excelsa, Macrolobium multijugum, Tachigali vaupesiana*). Although this was the highest number of monodominant species by family, the number was not higher (nor lower) than expected by chance based on the number of species of Fabaceae in all plots (780). Arecaceae species are among the most hyperdominant in Amazonia^32^ and had five monodominant species in the plots (*Mauritia flexuosa, Euterpe oleracea, Astrocaryum macrocalyx, Attalea speciosa, Astrocaryum murumuru*). The randomization tests suggested that the number of families found with monodominant species did not deviate from a random expectation, except in the case of dominance over 20% and over 80%. Subsequent tests with Bonferroni correction suggested that only Arecaceae and Bignoniaceae have more species with dominance higher than 20% and only Vochysiaceae higher than 80%. Thus with monodominance defined at 50% or higher no family has more monodominant species than expected by chance. Based on the tests with Bonferroni correction alone Arecaceae showed more often dominance from 20–50%. There was no consistent family pattern in the dominance classes of 60% and higher.

**Figure 2:**
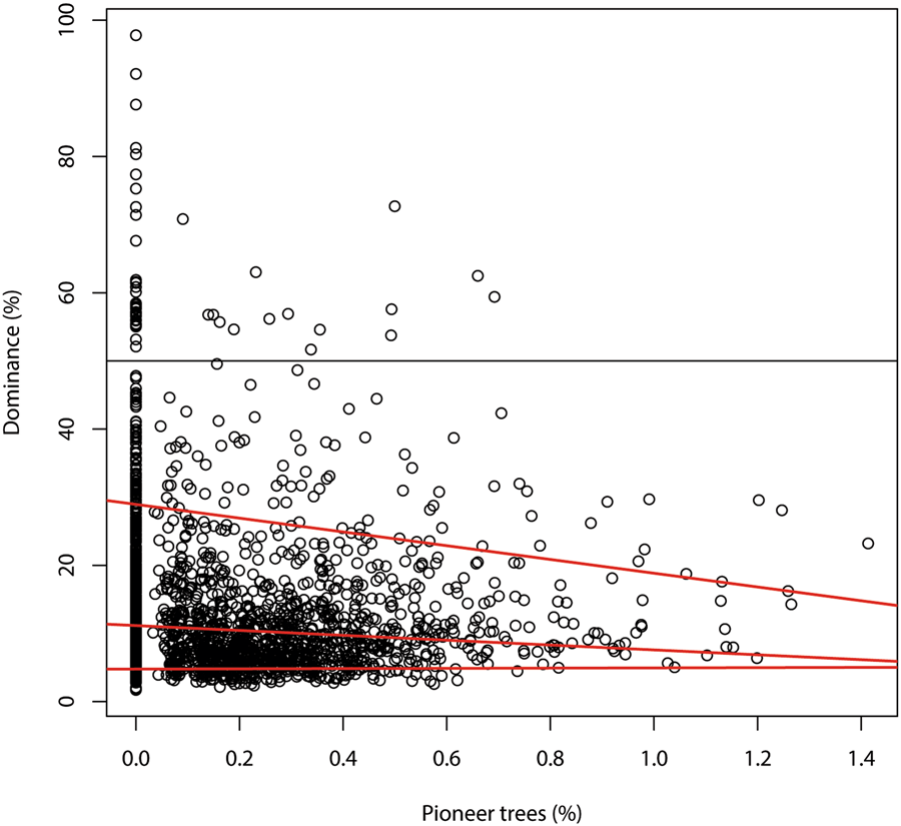
Dominance (= relative abundance of the most abundant species of each plot) by plot as a function of the percentage of pioneer trees in the plot. Lower red: Quantile regression line that separates the lower 10% from the upper 90% of the data (tau = 0.1, p = 0.34, i.e. slope not different from 0); middle red: quantile regression (tau = 0.5, p ≪ 0.001); upper red: quantile regression that separates the upper 10% of the data from the lower 90% (tau = 0.9, p = 0.035); black horizontal line: line of 50% dominance.

Dominance by plot appeared affected by the percentage of pioneer species (Fig. 2). While there was a weak (but significant) linear relationship between the two variables (p ≪ 0.001), maximum dominance appeared constrained more by a larger number of pioneers, as exemplified by a quantile regression for the upper 10% of the data (Tau = 0.9, p = 0.035), than did the average dominance (Fig. 2). Monodominance was found only on plots with less than 0.8% pioneers. However, this result was influenced by the low number of observations that had a high abundance of pioneers. We resampled the data 10,000 times taking 40 plots randomly from the ranges 0; 0–0.2; 0.2–0.4; 0.4–0.6; 0.6–0.8 and over 0.8% of pioneers. The average slope of the upper 10% quantile was -9.2, still showing a negative relationship but the 95% c.i. of the mean included also zero slope. Hence we could not detect a significant relationship between percentage of pioneers and maximum dominance.

**Figure 3:**
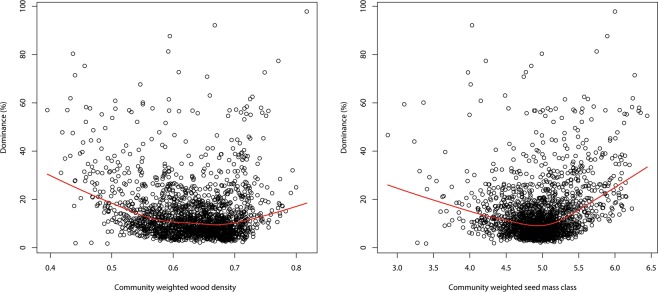
Dominance (= relative abundance of the most abundant species of each plot) at plot level as a function of community weighted wood density (**A**) and community weighted seed mass class (**B**). Red lines show a loess regression through the data.

Community weighted wood density and community weighted seed mass class had little effect but the average maximum dominance was highest with the lowest and highest values of each, consistent with the traits being part of the pioneer-climax continuum (Fig. 3). Monodominance was found in 14 genera, EM in ten, nodulation in 66, aluminium accumulation in 35, and coppicing in five (*Dimorphandra*, *Dicymbe, Euterpe, Pentaclethra*, and *Spirotropis*). The combination of monodominance and EM was found in *Dicymbe* (p = 0.16); monodominance and nodulation in the three genera *Machaerium*, *Spirotropis*, and *Tachigali* (p = 0.46); monodominance and aluminium accumulation in *Ruizterania* (p = 0.35), and monodominance and coppicing in two genera (*Dicymbe* and *Spirotropis*, p = 0.007). Thus, in our data, the only ecological trait significantly linked to monodominance was coppicing. On 201 plots one of the 85 recognized Amazonian domesticated species^69^ was the most abundant species (Appendix S3). In almost all cases (173 plots) and in all cases with a dominance over 30% this most dominant species was an Arecaceae species (Appendix S3). *Theobroma cacao* was the most dominant species on 10 plots.

**Figure 4:**
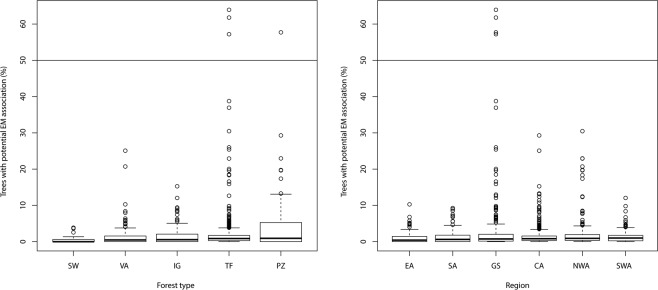
Percentage of trees belonging to potential ectomycorrhizal genera as a function of forest type (**A**) and region (**B**).

The highest percentages (outliers) of trees belonging to potential EM genera were found in white sand forest (PZ) and/or the Guiana Shield (Fig. 4). This forest type and region had both the highest median values as well as most of the high values for percentage EM. However, forest type explained only 2.6% (p ≪ 0.001) of the variation in EM percentage, and region 1.1% (p ≪ 0.001).

**Figure 5:**
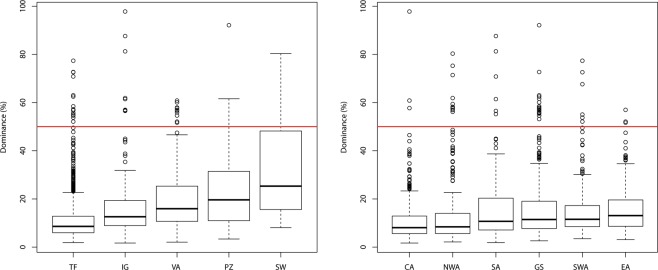
Percentage of dominance by plot (= relative abundance of the most abundant species of each plot) as a function of forest type (**A**) and region (**B**).

Maximum dominance was highest on those soil types with the smallest area in Amazonia (Fig. 5A). Each of the smaller forest types had higher median maximum dominance than *terra firme*. Forest type explained 28% (p ≪ 0.001) of the maximum dominance by plot (ANOVA). Median dominance was strongly related (power function) to area (Fig. S5). Region had only a very small effect (3% explained variation, p ≪ 0.001, Fig. 5B).

## Discussion

Monodominance (defined here as ≥50% of individuals ≥10 cm dbh in a stand belong to a single tree species) appears to be quite rare over the greater Amazonian region. In the ATDN analysis presented here, a very small percentage of all plots (2.6%) and species (0.5%) exhibited monodominance by the above definition, and even less, at 1.0% and 0.3%, respectively, under the definition of classical monodominance sensu Peh, *et al*.^15^. Even dominance between 20% and 50% was not common. The overall scarcity of monodominance at plot and species level may partially be a reflection of lack of specific sampling throughout the ATDN dataset. If tree plots were not set up to capture stands with clearly dominant tree species but rather set up to capture tree-diverse forest types, the dominant stands would be “missed”. Some ATDN plots, however, were set up to study monodominance, such as those dominated by *Brosimum rubescens*^13^, *Dicymbe corymbosa*^23^, *Spirotropis longifolia*^42^ and studies of plant communities in white sand systems of Guyana and Suriname^71^. In general we believe most plots were not selected on the basis of selecting or avoiding monodominance. Records do exist for some dominant tree species that were not confirmed as monodominant or were not captured in the ATDN plot data. For example, *Dicymbe altsonii* and *Dicymbe jenmanii* are each dominant to monodominant in parts of Guyana^46,72,73^, as well as *Pakaraimaea dipterocarpacea* in W. Guyana/E. Venezuela^72,74^, *Aldina* spp. in N. Brazil/S. Venezuela/W. Guyana^75–78^, and *Pseudomonotes tropenbosii* in E. Colombia (Aida Vasco-Palacios pers. comm.). All of these genera are confirmed EM^46,75^. Several ATDN plots have been established in peat swamps, the habitat type that probably accounts for the largest area of monodominant forests in Amazonia, within which only *Mauritia flexuosa* and *Pachira nitida* were able to attain monodominance^79^. *Micrandra* spp. (Euphorbiaceae) are also known to strongly dominate poorly-drained soils in W. Guyana^80,81^, T.W. Henkel pers. obs. and adjacent Venezuela^82^. All told, the overall ranking of dominance suggests a rather smooth transition across all dominance levels (Fig. 1).

Only two families had more species at dominance classes over 20% than expected by chance. The most consistent family with significant dominance (based on Bonferroni correction alone) from 20% to 50% is Arecaceae, including 20/74 species in our data. This is consistent with Arecaceae also having a five times higher than expected number of hyperdominant species^32^, reflecting their regularly high local dominance. Palms may reach high dominance because they are competitive in large wet areas but they must also be fairly resistant to frequency dependent mortality, as should other hyperdominant and monodominant species. For all other families the monodominance level is rather unpredictable, so we have no reason to suggest that certain families have a predisposition for monodominance in Amazonian forests.

Disturbance, as measured by its proxy pioneer abundance, and traits related to the pioneer-climax continuum had no significant effect on dominance or diversity, contrary to findings in an earlier Afro-tropical study in Ghana^38^ and a study of the effects of gap-scale disturbance in Amazonian forest that found a very small effect of disturbance on diversity or dominance^83^. Two French Guiana studies that used pioneer species as surrogates for disturbance regime found a stronger relationship^36,84^. Thus as in our data monodominance was only observed in plots with very low abundance of pioneers (Fig. 2), this was not a significant pattern.

In our analysis coppicing was the only trait significantly linked to monodominance. Coppicing occurs in many species after logging or clear felling but many coppices eventually die (HtS pers. obs.). Coppicing is not common as a natural means of regeneration and has been observed mainly in species of Fabaceae genera (*Dimorphandra*, *Dicymbe, Pentaclethra, Spirotropis*), one palm (*Euterpe oleracea*), *Humiria* and *Theobroma cacao*^85^. In *Dimorphandra* and *Humiria* coppicing is a rapid response to fire damage and species of each can become dominant in Guyana and Suriname in fire-prone savannah-forest ecotones^86^. In closed-canopy forest they are also found as non-coppicing tall trees. *Dicymbe* species exhibit both EM and very pronounced coppicing in the absence of mechanical disturbance, especially in *D. corymbosa*, and to a lesser extent *D. altsonii*^87^. Woolley, *et al*.^28^ hypothesised that the coppicing in *D. corymbosa* was an evolved response to persistent infections with heart-rot fungi, the adaptive significance being that the coppicing insures persistence of the individual beyond that which would occur with a heart-rotted, single-bole tree. Thus, while coppicing was observed as an important reproduction strategy for some Amazonian tree species or as a major regeneration process in secondary growth forests, none of the above can answer the obvious question as to why more species do not spontaneously coppice in mature forest.

Similar questions can be asked for the EM habit. Seedlings of species with access to an EM network may have higher survivorship, growth and reduced density-dependent mortality relative to AM trees^45^. Ectomycorrhizal associations may also provide a competitive edge by directly accessing organic forms of nutrients in litter, leaving little for saprotrophic fungi or AM mycorrhizae^88^. However, Mayor & Henkel (2006) used reciprocal litter transplants in *Dicymbe* monodominant forest and mixed AM-dominated forests and found no differences in litter decomposition rate between the forest types, or within the *Dicymbe* forest between trenched (EM-absent) and non-trenched (EM-present) plots. Conversely, McGuire, *et al*.^89^ found slower litter decomposition in monodominant *Dicymbe* forest, and lower richness of saprotrophic fungi than in adjacent mixed forest. Although EM has often been linked mechanistically to monodominance^9,43–46^, we did not find a significant relationship between monodominance and EM in the ATDN analysis. This contrasts with the review of Corrales, *et al*.^48^ in which both monodominance and confirmed mycorrhizal type were linked in both the Paleo- and Neotropics, and the majority of fully documented monodominant tree species were EM.

Neither nodulation nor aluminium accumulation were significantly related to monodominance. While N-fixing arguably should confer a large benefit on nitrogen-limited soils, none of the monodominant Fabaceae fix nitrogen, as is the case in the Fabaceae in the wet Afrotropics, where in contrast to Amazonia most dominant Fabaceae are EM e.g.^90^. At an Amazonia-wide scale Fabaceae dominance and N-fixing appear negatively correlated, and N-fixing Fabaceae do not dominate the most oligotrophic Amazonian ecosystems^56^. N-fixing is more prominent in forests richer in species^56^ and appears to have the greatest advantage in tropical dry successional forests^57^. Aluminium accumulation is found predominantly in a select number of families (e.g. Rubiaceae, Melastomataceae, Myrtaceae and Vochysiaceae; see references in Introduction) and one species (*Ruizteranea retusa*) was found as monodominant in our plots in southern Amazonia, while another monodominant species, *Vochysia divergens*, has been observed in the Brazilian Pantanal^62^. While aluminium accumulators are found abundantly in the Cerrado south of Amazonia e.g.^61^, they appear rare in wet Amazonian forests.

Although domesticated species were the most dominant species on 201 of the 1946 plots, in almost all cases these were Arecaceae, which tend to dominate large stretches of swamp forest in Amazonia (*Oenocarpus bataua, Euterpe oleracea, Mauritia flexuosa*) and in the case of monodominance only *Euterpe oleracea* and *Mauritia flexuosa*. With regard to *Mauritia flexuosa* this species had already attained high prominence in the Amazonian landscape prior to the arrival of humans^91,92^.

Area had a strong effect on dominance. The ‘forest type’ with the smallest areal coverage had by far the highest mean dominance (Fig. S5). It has been argued before that smaller ecosystems in Amazonia would have lower overall tree diversity and more dominant species^66,93^. This would be in line with ecological theories where equilibria of immigration and extinction maintain diversity^94,95^. Connell and Lowman^9^ noted that “*Single-species dominance is of less interest in regions that have smaller species pools*” and did “*not consider tropical forests at high altitudes, on small islands, or with low or very seasonal rainfall and/or extreme soil conditions, for example, frequently flooded freshwater swamps or mangrove forests, all habitats with few species*”. In the Amazon, however, this may be the most common road to monodominance. Nascimento, *et al*.^19^ also argued that drainage and other edaphic factors drive monodominance of *Peltogyne gracilipes* in one Amazonian forest. Similarly, Draper, *et al*.^79^ argued that the extreme environmental conditions of Amazonian peatland forests (waterlogging and low fertility), contributed to monodominance of *Mauritia flexuosa* and *Pachira nitida*. In the case of classical monodominance of the Congolian *G. dewevrei*, the discussion is ongoing. Kearsley, *et al*.^21^ suggested that “*environmental filtering prevailed in the monodominant G. dewevrei forest, leading to lower functional diversity in this forest type, with the dominant species showing beneficial traits related to its common riverine locations and with reduced soil N and P availability found in this environment, both co-regulating the tree community assembly*”. Others, however, found no edaphic differences between the monodominant *G. dewevrei* forest and adjacent mixed forest^25,26^, a result also found with monodominant *D. corymbosa* in Guyana^23,46^. Environmental filtering would also not explain the extreme monodominance of *G. dewevrei* over hundreds of km^2^ of upland area in the Congo region^11,96^.

We were unable to test for basal area monodominance here as the majority of plots in the ATDN lack stem diameter measurements. Instead we used the number of individuals as our metric to determine monodominance. While this is an easily available measure, others have taken basal area and estimates of above-ground biomass as metrics. Monodominant species can differ at the plot level, e.g. *D. corymbosa* can have less than 60% of all individuals ≥ 10 cm dbh in some plots, but exhibit 80–90% basal area dominance, due to its complete dominance of individuals in the very large size classes^23^. Such a stand is still most definitely monodominant, in terms of a single species commanding the majority of site resources, and in these same stands conspecifics will always be dominant in the seedling and sapling classes. Connell and Lowman^9^ pointed out that monodominance can be defined as a single species comprising >60% of individuals >10 cm dbh, or >60% of stand basal area, or both, and be considered “monodominant”.

While seed mass, shade tolerance, and longevity may theoretically lead to monodominance^15,16^, we find little support for these traits as being causal to monodominance. While monodominance can be mechanistically related to EM and coppicing, very few tree species have used these traits to dominate Amazonian forests. Large stands dominated by single species appear linked primarily to edaphic factors, such as swamps (e.g. many palm species), nutrient poor floodplains (*Eschweilera tenuifolia, Macrolobium, Triplaris, Symphonia*) and soils with poor drainage (*Micrandra* spp.)^80^, white sands (*Dicymbe, Eperua, Aldina*), soil chemical constraints (*Peltogyne, Brosimum*), or may be related to fire history (*Dimorphandra*).

In summary, we found that monodominance, as defined by stem abundance, is extremely rare in Amazonian tropical forests, at least within the extensive ATDN dataset, and found little support for a single mechanism for monodominance. The occurrence of monodominance was most strongly linked to metacommunity dynamics of small rare ecosystems, such as white sands, peats and flooded areas. Because the edaphic differences of the forest types with smaller fragmented areas (white sand forests, Várzea, Igapó and swamp forests) with the major forest type in the Amazon (terra firme), the “forest types” in Amazonia have their to a large extent a distinct tree flora. Within these areas dominance may be in part attributed to chance – the smaller and more fragmented the forest type area, the higher the chance for local dominance.

## Material and Methods

All tree data were derived from the Amazon Tree Diversity Network (ATDN, http://atdn.myspecies.info/), comprised of a long-term data set now containing >2000 tree inventory plots across Amazonia. Our analyses were based on 1946 plots, comprised of 127 families, 798 genera, and 5027 identified tree species. All analyses were performed using the R programming language^97^.

Firstly, dominance was calculated by plot. Dominance was defined as the relative abundance of the most abundant tree species within the community and was calculated as:

$$Dominance={N}_{d}/{N}_{tot}$$

where *N*_*d*_ is the number of individuals of the most abundant species and *N*_*tot*_ the total number of individuals in the tree plot (Dominance calculated this way is also known as the Berger-Parker index). We calculated rank dominance curves for dominance based on plots and mapped dominance across Amazonia. To study which families have more dominant species than expected by chance we listed all species with dominance over eight dominance classes (20–90%) by family. Then, with a Monte Carlo randomization test (1000 randomizations) we determined which tree families have more dominant/competitive species in each dominance class than expected by chance (based on the total number of tree species in the family). Maximum relative abundance of each species was also calculated, thus including species that were never the most dominant species in a plot. As we carried out as many tests as there are families at an error level of 5%, we can expect that at least 5% of the families may become false positives. We tested this by calculating for each of the 1000 randomizations how many families met this criterion and calculated mean and standard deviation. If the number of families found was significantly higher than this mean we applied Bonferroni correction (adjusting p as (p/number of families)), to find those families that were most likely to be the true positives of this test.

To test for competitive exclusion as a mechanism for dominance we used the percentage of pioneer species (log transformed to normalize the data) at plot level, as a proxy for disturbance^36,38,84,98,99^. We identified pioneers by combining low wood density and low seed mass under the condition (WD < 0.7 Λ SMC < 4, Fig. S2) sensu^99^. We used a loess regression to test for a relationship between the disturbance proxy and dominance at plot level.

To test if particular traits are linked to monodominance we examined two traits, wood density and seed mass, that are generally linked to longevity and dominance^39,100^. We calculated the community weighted average for both wood density and seed size as follows:

$$CWA=\sum {N}_{i}^{\ast }trait/\sum N$$

where C*WA* is the community weighted average, *∑N*_*i*_ is the sum of the number of individuals with trait data, *trait* is the corresponding trait value on genus level for either wood density or seed mass class and *∑N* is the total number of individuals in the tree community. We then carried out a loess regression to assess the relationship between the *CWA* of the functional traits and dominance.

To test if an EM association may lead to dominance we checked the most recent literature for confirmed EM tree species^101^. We tested if EM is more abundant on monodominant plots and if EM species are more likely to be monodominant. For a similar test for nodulation we used Sprent^102^ and Soltis, *et al*.^103^. For aluminium accumulation we used Jansen *et al*. (2002, 2003) and references therein. There is no single source for intrinsic coppicing, a means of persistence once an individual is established, in tropical trees - this information was collected from observations on our plots. We tested the association with Monte Carlo randomizations (n = 10,000).

To test if an area effect may lead to (mono-)dominance we used ANOVA to test if monodominance is more common in the forest types that have a smaller extent in Amazonia: white sand forest (4.6%)^104^, *igapó* and *várzea* (10%)^104,105^ and swamps (1.7%)^106^, compared to *terra firme* which covers most of the remaining area.

The datasets generated during and/or analysed during the current study are available from the corresponding author on reasonable request.

## Supplementary information

Supplementary information to Rarity of monodominance in hyperdiverse Amazonian forests

Supplementary Dataset 1
