# Supplementary material for: Rarity of monodominance in hyperdiverse Amazonian forests
Source: Sci Rep. 2019 Sep 25;9:13822. doi: 10.1038/s41598-019-50323-9 (PMC6761143; doi:10.1038/s41598-019-50323-9)
Supplement: Supplementary file 1 — Supplementary information to Rarity of monodominance in hyperdiverse Amazonian forests [file 41598_2019_50323_MOESM1_ESM.pdf]

Supplementary information to

Rarity of monodominance in hyperdiverse Amazonian forests

Hans ter Steege,1,2 Terry W. Henkel,3 Nora Helal,1 Beatriz S. Marimon,4 Ben Hur Marimon-Junior,4 Andreas Huth,5 Jürgen Groeneveld,5,6 Daniel Sabatier,7 Luiz de Souza Coelho,8 Diogenes de Andrade Lima Filho,8 Rafael P. Salomão,9,10 Iêda Leão Amaral,8 Francisca Dionízia de Almeida Matos,8 Carolina V. Castilho,11 Oliver L. Phillips,12 Juan Ernesto Guevara,13,14 Marcelo de Jesus Veiga Carim,15 Dairon Cárdenas López,16 William E. Magnusson,17 Florian Wittmann,18,19 Mariana Victória Irume,8 Maria Pires Martins,8 José Renan da Silva Guimarães,15 Jean-François Molino,7 Olaf S. Bánki,20 Maria Teresa Fernandez Piedade,21 Nigel C.A. Pitman,22 Abel Monteagudo Mendoza,23 José Ferreira Ramos,8 Bruno Garcia Luize,24 Evlyn Márcia Moraes de Leão Novo,25 Percy Núñez Vargas,26 Thiago Sanna Freire Silva,27 Eduardo Martins Venticinque,28 Angelo Gilberto Manzatto,29 Neidiane Farias Costa Reis,30 John Terborgh,31,32 Katia Regina Casula,30 Euridice N. Honorio Coronado,33,12 Juan Carlos Montero,34,8 Ted R. Feldpausch,35,12 Alvaro Duque,36 Flávia R.C. Costa,8 Nicolás Castaño Arboleda,16 Jochen Schöngart,21 Timothy J. Killeen,37 Rodolfo Vasquez,23 Bonifacio Mostacedo,38 Layon O. Demarchi,21 Rafael L. Assis,39 Chris Baraloto,40 Julien Engel,7,40 Pascal Petronelli,41 Hernán Castellanos,42 Marcelo Brilhante de Medeiros,43 Adriano Quaresma,21 Marcelo Fragomeni Simon,43 Ana Andrade,44 José Luís Camargo,44 Susan G.W. Laurance,32 William F. Laurance,32 Lorena M. Rincón,8 Juliana Schietti,8 Thaiane R. Sousa,8 Emanuelle de Sousa Farias,45,46 Maria Aparecida Lopes,47 José Leonardo Lima Magalhães,48,49 Henrique Eduardo Mendonça Nascimento,8 Helder Lima de Queiroz,50 Gerardo A. Aymard C.,51 Roel Brienen,12 Juan David Cardenas Revilla,8 Ima Célia Guimarães Vieira,10 Bruno Barçante Ladvocat Cintra,21,12 Pablo R. Stevenson,52 Yuri Oliveira Feitosa,53 Joost F. Duivenvoorden,54 Hugo F. Mogollón,55 Alejandro Araujo-Murakami,56 Leandro Valle Ferreira,10 José Rafael Lozada,57 James A. Comiskey,58,59 José Julio de Toledo,60 Gabriel Damasco,61 Nállarett Dávila,62 Freddie Draper,63,40 Roosevelt García-Villacorta,64,65 Aline Lopes,21,66 Alberto Vicentini,17 Alfonso Alonso,59 Francisco Dallmeier,59 Vitor H.F. Gomes,10,67 Jon Lloyd,68 David Neill,69 Daniel Praia Portela de Aguiar,21 Luzmila Arroyo,56 Fernanda Antunes Carvalho,17,70 Fernanda Coelho de Souza,17,12 Dário Dantas do Amaral,10 Kenneth J. Feeley,71,72 Rogerio Gribel,73 Marcelo Petratti Pansonato,8,74 Jos Barlow,75 Erika Berenguer,76 Joice Ferreira,49 Paul V.A. Fine,61 Marcelino Carneiro Guedes,77 Eliana M. Jimenez,78 Juan Carlos Licona,34 Maria Cristina Peñuela Mora,79 Boris Villa,21 Carlos Cerón,80 Paul Maas,81 Marcos Silveira,82 Juliana Stropp,83 Raquel Thomas,84 Tim R. Baker,12 Doug Daly,85 Kyle G. Dexter,86,65 Isau Huamantupa-Chuquimaco,26 William Milliken,87 Toby Pennington,35,65 Marcos Ríos Paredes,88 Alfredo Fuentes,89,90 Bente Klitgaard,91 José Luis Marcelo Pena,92 Carlos A. Peres,93 Miles R. Silman,94 J. Sebastián Tello,90 Jerome Chave,95 Fernando Cornejo Valverde,96 Anthony Di Fiore,97 Renato Richard Hilário,60 Juan Fernando Phillips,98 Gonzalo Rivas-Torres,99,100 Tinde R. van Andel,1 Patricio von Hildebrand,101 Janaína Costa Noronha,102 Edelcilio Marques Barbosa,8 Flávia Rodrigues Barbosa,102 Luiz Carlos de Matos Bonates,8 Rainiellen de Sá Carpanedo,102 Hilda Paulette Dávila Doza,88 Émile Fonty,103,7 Ricardo Zárate Gómez,104 Therany Gonzales,105 George Pepe Gallardo Gonzales,88 Bruce Hoffman,106 André Braga Junqueira,107 Yadvinder Malhi,108 Ires Paula de Andrade Miranda,8 Linder Felipe Mozombite Pinto,88 Adriana Prieto,109 Domingos de Jesus Rodrigues,102 Agustín Rudas,109 Ademir R. Ruschel,49 Natalino Silva,110 César I.A. Vela,111 Vincent Antoine Vos,112 Egleé L. Zent,113 Stanford Zent,113 Bianca Weiss Albuquerque,21 Angela Cano,52,114 Yrma Andreina Carrero Márquez,57 Diego F. Correa,52,115 Janaina Barbosa Pedrosa Costa,77 Bernardo Monteiro Flores,116 David Galbraith,12 Milena Holmgren,117 Michelle Kalamandeen,12 Marcelo Trindade Nascimento,118 Alexandre A. Oliveira,74 Hirma Ramirez-Angulo,119 Maira Rocha,21 Veridiana Vizoni Scudeller,120 Rodrigo Sierra,121 Milton Tirado,121 Maria Natalia Umaña Medina,52,122 Geertje van der Heijden,123 Emilio Vilanova Torre,119,124 Corine Vriesendorp,22 Ophelia Wang,125 Kenneth R. Young,126 Manuel Augusto Ahuite Reategui,127 Cláudia Baider,128,74 Henrik Balslev,129 Sasha Cárdenas,52 Luisa Fernanda Casas,52 William Farfan-Rios,94 Cid Ferreira,8 Reynaldo Linares-Palomino,59 Casimiro Mendoza,130,131 Italo Mesones,61 Armando Torres-Lezama,119 Ligia Estela Urrego Giraldo,36 Daniel Villarroel,56 Roderick Zagt,132 Miguel N. Alexiades,133 Edmar Almeida de Oliveira,4 Karina

Garcia-Cabrera,94 Lionel Hernandez,42 Walter Palacios Cuenca,134 Susamar Pansini,30 Daniela Pauletto,135 Freddy Ramirez Arevalo,136 Adeilza Felipe Sampaio,30 Elvis H. Valderrama Sandoval,137,136 Luis Valenzuela Gamarra,23 Aurora Levesley,12 Georgia Pickavance,12 Karina Melgaço,12

1Biodiversity Dynamics, Naturalis Biodiversity Center, PO Box 9517, Leiden, 2300 RA, The Netherlands
2Systems Ecology, Free University, De Boelelaan 1087, Amsterdam, 1081 HV, The Netherlands
3Department of Biological Sciences, Humboldt State University, 1 Harpst Street, Arcata, CA, 95521, USA
4Programa de Pós-Graduação em Ecologia e Conservação, Universidade do Estado de Mato Grosso, Nova Xavantina, MT, Brazil
5Department of Ecological Modelling, Helmholtz Centre for Environmental Research - UFZ, Permoserstr. 15, Leipzig, 4318, Germany
6Institute of Forest Growth and Computer Sciences, Technische Universitaet Dresden, Postfach 1117, Tharandt, 1735, Germany
7AMAP, IRD, Cirad, CNRS, INRA, Université de Montpellier, TA A-51/PS2, Bd. de la Lironde, Montpellier, F-34398, France. A comprehensive
8Coordenação de Biodiversidade, Instituto Nacional de Pesquisas da Amazônia - INPA, Av. André Araújo, 2936, Petrópolis, Manaus, AM, 69067-375, Brazil
9Programa Professor Visitante Nacional Sênior na Amazônia - CAPES, Universidade Federal Rural da Amazônia, Av. Perimetral, s/n, Belém, PA, Brazil
10Coordenação de Botânica, Museu Paraense Emílio Goeldi, Av. Magalhães Barata 376, C.P. 399, Belém, PA, 66040-170, Brazil
11EMBRAPA – Centro de Pesquisa Agroflorestal de Roraima, BR 174, km 8 – Distrito Industrial, Boa Vista, RR, 69301-970, Brazil
12School of Geography, University of Leeds, Woodhouse Lane, Leeds, LS2 9JT, UK
13Grupo de Investigación en Biodiversidad, Medio Ambiente y Salud-BIOMAS, Universidad de las Américas, Campus Queri, Quito, Ecuador
14Keller Science Action Center, The Field Museum, 1400 S. Lake Shore Drive, Chicago, IL, 60605-2496, USA
15Departamento de Botânica, Instituto de Pesquisas Científicas e Tecnológicas do Amapá - IEPA, Rodovia JK, Km 10, Campus do IEPA da Fazendinha, Amapá, 68901-025, Brazil
16Herbario Amazónico Colombiano, Instituto SINCHI, Calle 20 No 5-44, Bogotá, DC, Colombia
17Coordenação de Pesquisas em Ecologia, Instituto Nacional de Pesquisas da Amazônia - INPA, Av. André Araújo, 2936, Petrópolis, Manaus, AM, 69067-375, Brazil
18Dep. of Wetland Ecology, Institute of Geography and Geoecology, Karlsruhe Institute of Technology - KIT, Josefstr.1, Rastatt, D-76437, Germany
19Biogeochemistry, Max Planck Institute for Chemistry, Hahn-Meitner Weg 1, Mainz, 55128, Germany
20Naturalis Biodiversity Center, PO Box 9517, Leiden, 2300 RA, The Netherlands
21Coordenação de Dinâmica Ambiental, Instituto Nacional de Pesquisas da Amazônia - INPA, Av. André Araújo, 2936, Petrópolis, Manaus, AM, 69067-375, Brazil
22Science and Education, The Field Museum, 1400 S. Lake Shore Drive, Chicago, IL, 60605-2496, USA
23Jardín Botánico de Missouri, Oxapampa, Pasco, Peru
24Departamento de Ecologia, Universidade Estadual Paulista - UNESP – Instituto de Biociências – IB, Av. 24 A, 1515, Bela Vista, Rio Claro, SP, 13506-900, Brazil
25Divisao de Sensoriamento Remoto – DSR, Instituto Nacional de Pesquisas Espaciais – INPE, Av. dos Astronautas, 1758, Jardim da Granja, São José dos Campos, SP, 12227-010, Brazil
26Herbario Vargas, Universidad Nacional de San Antonio Abad del Cusco, Avenida de la Cultura, Nro 733, Cusco, Cuzco, Peru
27Departamento de Geografia, Universidade Estadual Paulista -UNESP – Instituto de Geociências e Ciências Extas – IGCE, Bela Vista, Rio Claro, SP, 13506-900, Brazil
28Centro de Biociências, Departamento de Ecologia, Universidade Federal do Rio Grande do Norte, Av. Senador Salgado Filho, 3000 , Natal, RN, 59072-970, Brazil
29Departamento de Biologia, Universidade Federal de Rondônia, Rodovia BR 364 s/n Km 9,5 - Sentido Acre, Unir, Porto Velho, RO, 76.824-027, Brazil
30Programa de Pós- Graduação em Biodiversidade e Biotecnologia PPG- Bionorte, Universidade Federal de Rondônia, Campus Porto Velho Km 9,5 bairro Rural, Porto Velho, RO, 76.824-027, Brazil
31Department of Biology and Florida Museum of Natural History, University of Florida, Gainesville, FL, 32611, USA
32Centre for Tropical Environmental and Sustainability Science and College of Science and Engineering, James Cook University, Cairns, Queensland, 4870, Australia
33Instituto de Investigaciones de la Amazonía Peruana (IIAP), Av. A. Quiñones km 2,5, Iquitos, Loreto, 784, Peru
34Instituto Boliviano de Investigacion Forestal, Av. 6 de agosto #28, Km. 14, Doble via La Guardia, Casilla 6204, Santa Cruz, Santa Cruz, Bolivia
35Geography, College of Life and Environmental Sciences, University of Exeter, Rennes Drive, Exeter, EX4 4RJ, UK
36Departamento de Ciencias Forestales, Universidad Nacional de Colombia, Calle 64 x Cra 65, Medellín, Antioquia, 1027, Colombia
37Agteca-Amazonica, Santa Cruz, Bolivia
38Facultad de Ciencias Agrícolas, Universidad Autónoma Gabriel René Moreno, Santa Cruz, Santa Cruz, Bolivia
39Natural History Museum, University of Oslo, Postboks 1172, Oslo, 318, Norway
40International Center for Tropical Botany (ICTB) Department of Biological Sciences, Florida International University, 11200 SW 8th Street, OE 243, Miami, FL, 33199, USA
41Cirad UMR Ecofog, AgrosParisTech,CNRS,INRA,Univ Guyane, Campus agronomique, Kourou Cedex, 97379, France
42Centro de Investigaciones Ecológicas de Guayana, Universidad Nacional Experimental de Guayana, Calle Chile, urbaniz Chilemex, Puerto Ordaz, Bolivar, Venezuela
43Prédio da Botânica e Ecologia, Embrapa Recursos Genéticos e Biotecnologia, Parque Estação Biológica, Av. W5 Norte, Brasilia, DF, 70770-917, Brazil
44Projeto Dinâmica Biológica de Fragmentos Florestais, Instituto Nacional de Pesquisas da Amazônia - INPA, Av. André Araújo, 2936, Petrópolis, Manaus, AM, 69067-375, Brazil
45Laboratório de Ecologia de Doenças Transmissíveis da Amazônia (EDTA), Instituto Leônidas e Maria Deane, Fiocruz, Rua Terezina, 476, Adrianópolis, Manaus, AM, 69060-001, Brazil
46Programa de Pós-graduação em Biodiversidade e Saúde, Instituto Oswaldo Cruz - IOC/FIOCRUZ, Pav. Arthur Neiva – Térreo, Av. Brasil, 4365 – Manguinhos, Rio de Janeiro, RJ, 21040-360, Brazil

47Instituto de Ciências Biológicas, Universidade Federal do Pará, Av. Augusto Corrêa 01, Belém, PA, 66075-110, Brazil
48Programa de Pós-Graduação em Ecologia, Universidade Federal do Pará, Av. Augusto Corrêa 01, Belém, PA, 66075-110, Brazil
49Embrapa Amazônia Oriental, Trav. Dr. Enéas Pinheiro s/nº, Belém, PA, 66095-100, Brazil
50Diretoria Técnico-Científica, Instituto de Desenvolvimento Sustentável Mamirauá, Estrada do Bexiga, 2584, Tefé, AM, 69470-000, Brazil
51Programa de Ciencias del Agro y el Mar, Herbario Universitario (PORT), UNELLEZ-Guanare, Guanare, Portuguesa, 3350, Venezuela
52Laboratorio de Ecología de Bosques Tropicales y Primatología, Universidad de los Andes, Carrera 1 # 18a- 10, Bogotá, DC, 111711, Colombia
53Programa de Pós-Graduação em Biologia (Botânica), Instituto Nacional de Pesquisas da Amazônia - INPA, Av. André Araújo, 2936, Petrópolis, Manaus, AM, 69067-375, Brazil
54Institute of Biodiversity and Ecosystem Dynamics, University of Amsterdam, Sciencepark 904, Amsterdam, 1098 XH, The Netherlands
55Endangered Species Coalition, 8530 Geren Rd., Silver Spring, MD, 20901, USA
56Museo de Historia Natural Noel Kempff Mercado, Universidad Autónoma Gabriel Rene Moreno, Avenida Irala 565 Casilla Post al 2489, Santa Cruz, Santa Cruz, Bolivia
57Facultad de Ciencias Forestales y Ambientales, Instituto de Investigaciones para el Desarrollo Forestal, Universidad de los Andes, Via Chorros de Milla, 5101, Mérida, Mérida, Venezuela
58Inventory and Monitoring Program, National Park Service, 120 Chatham Lane, Fredericksburg, VA, 22405, USA
59Center for Conservation and Sustainability, Smithsonian Conservation Biology Institute, 1100 Jefferson Dr. SW, Suite 3123, Washington, DC, 20560-0705, USA
60Universidade Federal do Amapá, Ciências Ambientais, Rod. Juscelino Kubitschek km2, Macapá, AP, 68902-280, Brazil
61Department of Integrative Biology, University of California, Berkeley, CA, 94720-3140, USA
62Biologia Vegetal, Universidade Estadual de Campinas, Caixa Postal 6109, Campinas, SP, 13.083-970, Brazil
63Department of Global Ecology, Carnegie Institution for Science, 260 Panama St., Stanford, CA, 94305, USA
64Institute of Molecular Plant Sciences, University of Edinburgh, Mayfield Rd, Edinburgh, EH3 5LR, UK
65Tropical Diversity Section, Royal Botanic Garden Edinburgh, 20a Inverleith Row, Edinburgh, Scotland, EH3 5LR, UK
66Department of Ecology, University of Brasilia, Brasilia, DF, 70904-970, Brazil
67Programa de Pós-Graduação em Ciência Ambientais, Universidade Federal do Pará, Rua Augusto Corrêa 01, Belém, PA, 66075-110, Brazil
68Faculty of Natural Sciences, Department of Life Sciences, Imperial College London, Silwood Park, South Kensington Campus, London, SW7 2AZ, UK
69Ecosistemas, Biodiversidad y Conservación de Especies, Universidad Estatal Amazónica, Km. 2 1/2 vía a Tena (Paso Lateral), Puyo, Pastaza, Ecuador
70Universidade Federal de Minas Gerais, Instituto de Ciências Biológicas, Departamento de Genética, Ecologia e Evolução, Av. Antônio Carlos, 6627 Pampulha, Belo Horizonte, MG, 31270-901, Brazil
71Department of Biology, University of Miami, Coral Gables, FL, 33146, USA
72Fairchild Tropical Botanic Garden, Coral Gables, FL, 33156, USA
73Diretoria de Pesquisas Científicas, Instituto de Pesquisas Jardim Botânico do Rio de Janeiro, Rio de Janeiro, RJ, Brazil
74Instituto de Biociências - Dept. Ecologia, Universidade de Sao Paulo - USP, Rua do Matão, Trav. 14, no. 321, Cidade Universitária, São Paulo, SP, 05508-090, Brazil
75Lancaster Environment Centre, Lancaster University, Lancaster, Lancashire, LA1 4YQ, UK
76Environmental Change Institute, University of Oxford, Oxford, Oxfordshire, OX1 3QY, UK
77Empresa Brasileira de Pesquisa Agropecuária, Embrapa Amapá, Rod. Juscelino Kubitschek km 5, Macapá, Amapá, 68903-419, Brazil
78Grupo de Investigación en Tecnologías de la Información y Medio Ambiente, Instituto Tecnológico de Antioquia - Institución Universitaria, Calle 78B No. 72A-220, Medellín, Colombia
79Universidad Regional Amazónica IKIAM, Km 7 via Muyuna, Tena, Napo, Ecuador
80Escuela de Biología Herbario Alfredo Paredes, Universidad Central, Ap. Postal 17.01.2177, Quito, Pichincha, Ecuador
81Taxonomy and Systematics, Naturalis Biodiversity Center, PO Box 9517, Leiden, 2300 RA, The Netherlands
82Museu Universitário / Centro de Ciências Biológicas e da Natureza / Laboratório de Botânica e Ecologia Vegetal, Universidade Federal do Acre, Rio Branco, AC, 69915-559, Brazil
83Institute of Biological and Health Sciences, Federal University of Alagoas, Av. Lourival Melo Mota, s/n, Tabuleiro do Martins, Maceio, AL, 57072-970, Brazil
84Iwokrama International Centre for Rainforest Conservation, Georgetown, Guyana
85New York Botanical Garden, 2900 Southern Blvd, Bronx, New York, NY, 10458-5126, USA
86School of Geosciences, University of Edinburgh, 201 Crew Building, King's Buildings, Edinburgh, EH9 3JN, UK
87Natural Capital and Plant Health, Royal Botanic Gardens, Kew, Richmond, Surrey, TW9 3AB, UK
88Servicios de Biodiversidad EIRL, Jr. Independencia 405, Iquitos, Loreto, 784, Peru
89Herbario Nacional de Bolivia, Universitario UMSA, Casilla 10077 Correo Central, La Paz, La Paz, Bolivia
90Center for Conservation and Sustainable Development, Missouri Botanical Garden, P.O. Box 299, St. Louis, MO, 63166-0299, USA
91Department for Identification & Naming, Royal Botanic Gardens, Kew, Richmond, Surrey, TW9 3AB, UK
92Department of Forestry Management, Universidad Nacional Agraria La Molina, Avenido La Molina, Apdo. 456, La Molina, Lima, Peru
93School of Environmental Sciences, University of East Anglia, Norwich, NR4 7TJ, UK
94Biology Department and Center for Energy, Environment and Sustainability, Wake Forest University, 1834 Wake Forest Rd, Winston Salem, NC, 27106, USA
95Laboratoire Evolution et Diversité Biologique, CNRS and Université Paul Sabatier, UMR 5174 EDB, Toulouse, 31000, France
96Andes to Amazon Biodiversity Program, Madre de Dios, Madre de Dios, Peru
97Department of Anthropology, University of Texas at Austin, SAC 5.150, 2201 Speedway Stop C3200, Austin, TX, 78712, USA
98Fundación Puerto Rastrojo, Cra 10 No. 24-76 Oficina 1201, Bogotá, DC, Colombia
99Colegio de Ciencias Biológicas y Ambientales-COCIBA & Galapagos Institute for the Arts and Sciences-GAIAS, Universidad San Francisco de Quito-USFQ, Quito, Pichincha, Ecuador
100Department of Wildlife Ecology and Conservation, University of Florida, 110 Newins-Ziegler Hall, Gainesville, FL, 32611, USA
101Fundación Estación de Biología, Cra 10 No. 24-76 Oficina 1201, Bogotá, DC, Colombia
102ICNHS, Federal University of Mato Grosso, Av. Alexandre Ferronato 1200, Setor Industrial, Sinop, MT, 78.557-267, Brazil
103Direction régionale de la Guyane, ONF, Cayenne, F-97300, French Guiana

104PROTERRA, Instituto de Investigaciones de la Amazonía Peruana (IIAP), Av. A. Quiñones km 2,5, Iquitos, Loreto, 784, Peru
105ACEER Foundation, Jirón Cusco N° 370, Puerto Maldonado, Madre de Dios, Peru
106Amazon Conservation Team, Doekhieweg Oost #24, Paramaribo, Suriname
107Institut de Ciència i Tecnologia Ambientals, Universitat Autònoma de Barcelona, 08193 Bellaterra, Barcelona, Spain
108Environmental Change Institute, Oxford University Centre for the Environment, Dyson Perrins Building, South Parks Road, Oxford, England, OX1 3QY, UK
109Instituto de Ciencias Naturales, Universidad Nacional de Colombia, Apartado 7945, Bogotá, DC, Colombia
110Instituto de Ciência Agrárias, Universidade Federal Rural da Amazônia, Av. Presidente Tancredo Neves 2501, Belém, PA, 66.077-830, Brazil
111Escuela Profesional de Ingeniería Forestal, Universidad Nacional de San Antonio Abad del Cusco, Jirón San Martín 451, Puerto Maldonado, Madre de Dios, Peru
112Universidad Autónoma del Beni José Ballivián, Campus Universitario Final, Av. Ejercito, Riberalta, Beni, Bolivia
113Laboratory of Human Ecology, Instituto Venezolano de Investigaciones Científicas - IVIC, Ado 20632, Caracas, Caracas, 1020A, Venezuela
114Cambridge University Botanic Garden, 1 Brookside., Cambridge, CB2 1JE, UK
115School of Agriculture and Food Sciences - ARC Centre of Excellence for Environmental Decisions CEED, The University of Queensland, St. Lucia, QLD 4072, Australia
116University of Campinas, Plant Biology Department, Rua Monteiro Lobato, 255, Cidade Universitária Zeferino Vaz, Barão Geraldo, Campinas, São Paulo, CEP 13083-862, Brazil
117Resource Ecology Group, Wageningen University & Research, Droevendaalsesteeg 3a, Lumen, building number 100, Wageningen, Gelderland, 6708 PB, The Netherlands
118Laboratório de Ciências Ambientais, Universidade Estadual do Norte Fluminense, Av. Alberto Lamego 2000, Campos dos Goyatacazes, RJ, 28013-620, Brazil
119Instituto de Investigaciones para el Desarrollo Forestal (INDEFOR), Universidad de los Andes, Conjunto Forestal, 5101, Mérida, Mérida, Venezuela
120Departamento de Biologia, Universidade Federal do Amazonas - UFAM – Instituto de Ciências Biológicas – ICB1, Av General Rodrigo Octavio 6200, Manaus, AM, 69080-900, Brazil
121GeoIS, El Día 369 y El Telégrafo, 3° Piso, Quito, Pichincha, Ecuador
122Department of Biology, University of Maryland, College Park, MD, 20742, USA
123University of Nottingham, University Park, Nottingham, NG7 2RD, UK
124School of Environmental and Forest Sciences, University of Washington, Seattle, WA, 98195-2100, USA
125Environmental Science and Policy, Northern Arizona University, Flagstaff, AZ, 86011, USA
126Geography and the Environment, University of Texas at Austin, 305 E. 23rd Street, CLA building, Austin, TX, 78712, USA
127Medio Ambiente, PLUSPRETOL, Iquitos, Loreto, Peru
128The Mauritius Herbarium, Agricultural Services, Ministry of Agro-Industry and Food Security, Reduit, 80835, Mauritius
129Department of Bioscience, Aarhus University, Building 1540 Ny Munkegade, Aarhus C, Aarhus, DK-8000, Denmark
130FOMABO, Manejo Forestal en las Tierras Tropicales de Bolivia, Sacta, Cochabamba, Bolivia
131Escuela de Ciencias Forestales (ESFOR), Universidad Mayor de San Simon (UMSS), Sacta, Cochabamba, Bolivia
132Tropenbos International, Lawickse Allee 11 PO Box 232, Wageningen, 6700 AE, The Netherlands
133School of Anthropology and Conservation, University of Kent, Marlowe Building, Canterbury, Kent, CT2 7NR, UK
134Herbario Nacional del Ecuador, Universidad Técnica del Norte, Quito, Pichincha, Ecuador
135Instituto de Biodiversidade e Floresta, Universidade Federal do Oeste do Pará, Rua Vera Paz, Campus Tapajós, Santarém, PA, 68015-110, Brazil
136Facultad de Biologia, Universidad Nacional de la Amazonia Peruana, Pevas 5ta cdra, Iquitos, Loreto, Peru
137Department of Biology, University of Missouri, St. Louis, MO, 63121, USA

Correspondence and requests for materials should be addressed to H.t.S. (email: hans.tersteege@naturalis.nl)

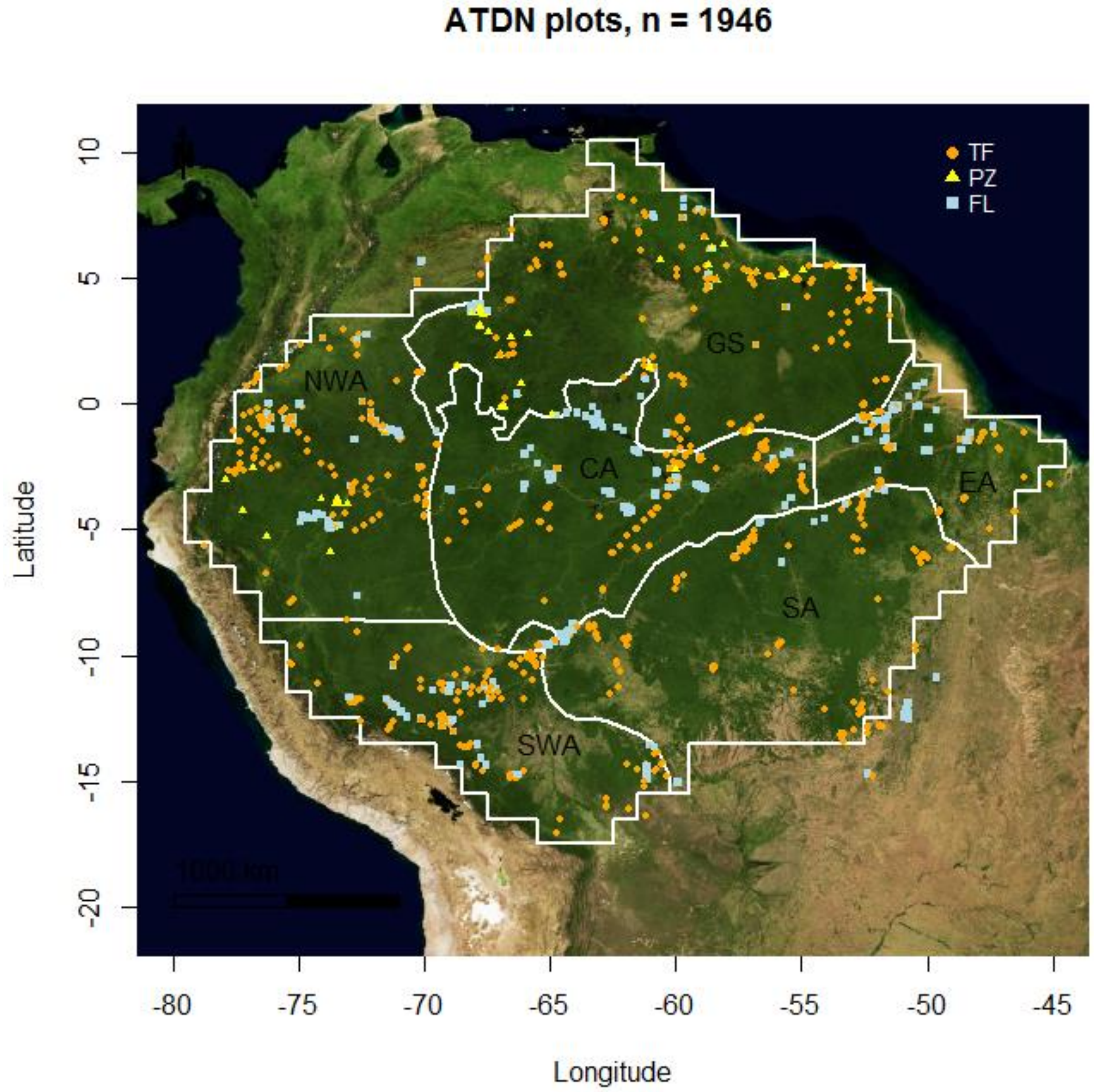

Figure S1. Map of Amazonia showing the location of the 1,946 Amazon Tree Diversity Network (ATDN) plots that contributed data to this paper. The white polygon marks our delimitation of the study area and consists of 567 1° grid cells (area = 6.29 million km2). Orange circles indicate plots on *terra firme*; blue squares, plots on seasonally or permanently flooded terrain (*várzea, igapó*, swamps); yellow triangles, plots on white-sand podzols; gray circles, plots only used for tree density calculations. Background is from Visible Earth. Regions: CA, central Amazonia; EA, eastern Amazonia; GS, Guiana Shield; SA, southern Amazonia; WAN, northwestern Amazonia; WAS, southwestern Amazonia. Map created with custom R[1] script. Base map source (country.shp, rivers.shp): ESRI (http://www.esri.com/data/basemaps, © Esri, DeLorme Publishing Company). Background: Visible Earth NASA [2] (https://visibleearth.nasa.gov/useterms.php © NASA).

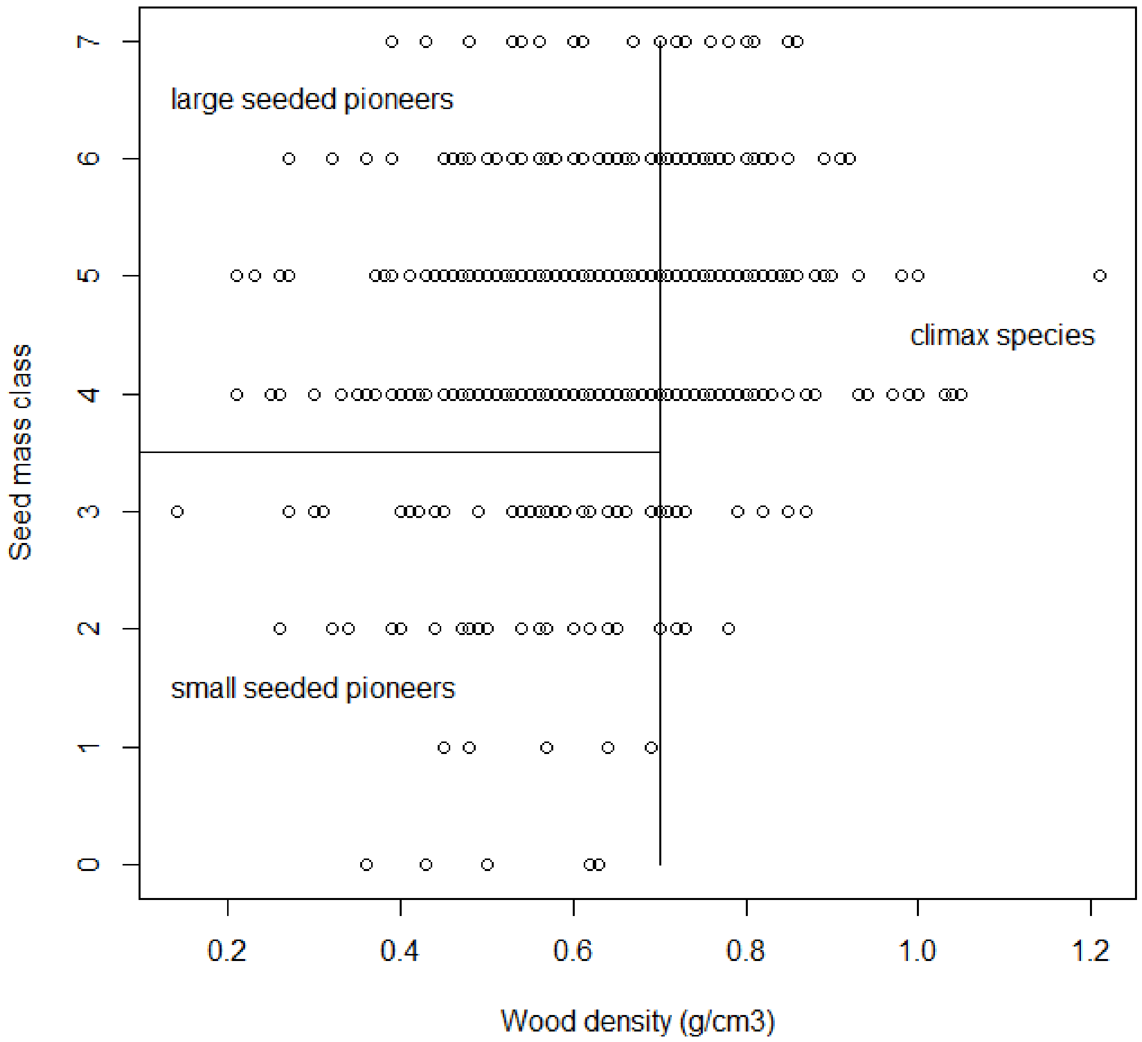

Figure S2. Wood density and seed mass class of all genera found in the plots used for this study.

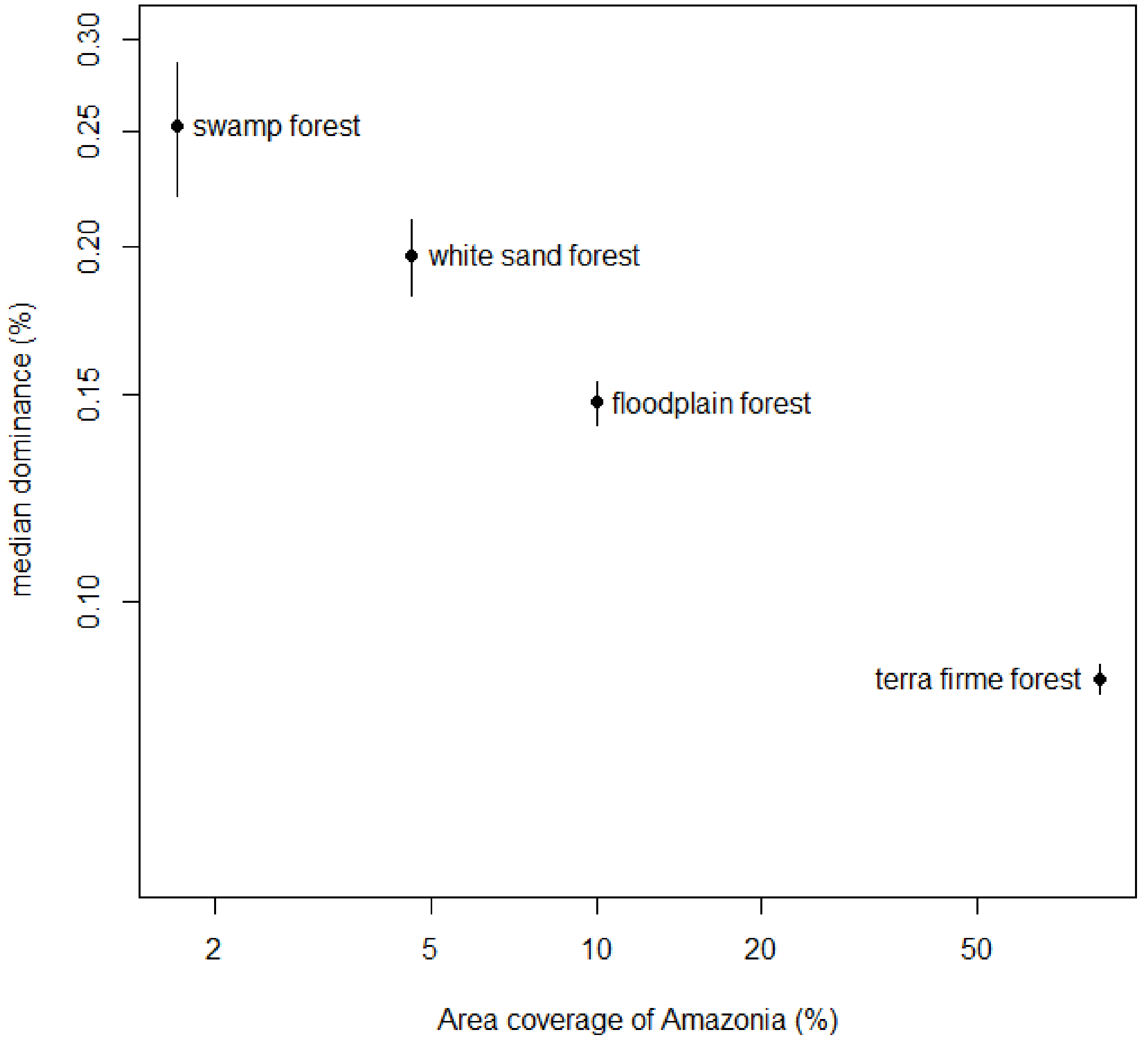

Figure S5. Median dominance of the four main Amazonian forest types by surface area coverage of Amazonia. Bars indicate se (sd/sqrt(n)).

References

1 R: A language and environment for statistical computing (R Foundation for Statistical Computing, Vienna, Austria, 2017).

2 Stöckli, R., Vermote, E., Saleous, N., Simmon, R. & Herring, D. The Blue Marble Next Generation - A true color earth dataset including seasonal dynamics from MODIS. (NASA Earth Observatory, 2005).

Consortia

ATDN (Amazon Tree Diversity Network)

Luiz de Souza Coelho,8 Diogenes de Andrade Lima Filho,8 Rafael P. Salomão,9,10 Iêda Leão Amaral,8 Francisca Dionízia de Almeida Matos,8 Carolina V. Castilho,11 Oliver L. Phillips,12 Juan Ernesto Guevara,13,14 Marcelo de Jesus Veiga Carim,15 Dairon Cárdenas López,16 William E. Magnusson,17 Florian Wittmann,18,19 Mariana Victória Irume,8 Maria Pires Martins,8 José Renan da Silva Guimarães,15 Jean-François Molino,7 Olaf S. Bánki,20 Maria Teresa Fernandez Piedade,21 Nigel C.A. Pitman,22 Abel Monteagudo Mendoza,23 José Ferreira Ramos,8 Bruno Garcia Luize,24 Evlyn Márcia Moraes de Leão Novo,25 Percy Núñez Vargas,26 Thiago Sanna Freire Silva,27 Eduardo Martins Venticinque,28 Angelo Gilberto Manzatto,29 Neidiane Farias Costa Reis,30 John Terborgh,31,32 Katia Regina Casula,30 Euridice N. Honorio Coronado,33,12 Juan Carlos Montero,34,8 Ted R. Feldpausch,35,12 Alvaro Duque,36 Flávia R.C. Costa,8 Nicolás Castaño Arboleda,16 Jochen Schöngart,21 Timothy J. Killeen,37 Rodolfo Vasquez,23 Bonifacio Mostacedo,38 Layon O. Demarchi,21 Rafael L. Assis,39 Chris Baraloto,40 Julien Engel,7,40 Pascal Petronelli,41 Hernán Castellanos,42 Marcelo Brilhante de Medeiros,43 Adriano Quaresma,21 Marcelo Fragomeni Simon,43 Ana Andrade,44 José Luís Camargo,44 Susan G.W. Laurance,32 William F. Laurance,32 Lorena M. Rincón,8 Juliana Schietti,8 Thaiane R. Sousa,8 Emanuelle de Sousa Farias,45,46 Maria Aparecida Lopes,47 José Leonardo Lima Magalhães,48,49 Henrique Eduardo Mendonça Nascimento,8 Helder Lima de Queiroz,50 Gerardo A. Aymard C.,51 Roel Brienen,12 Juan David Cardenas Revilla,8 Ima Célia Guimarães Vieira,10 Bruno Barçante Ladvocat Cintra,21,12 Pablo R. Stevenson,52 Yuri Oliveira Feitosa,53 Joost F. Duivenvoorden,54 Hugo F. Mogollón,55 Alejandro Araujo-Murakami,56 Leandro Valle Ferreira,10 José Rafael Lozada,57 James A. Comiskey,58,59 José Julio de Toledo,60 Gabriel Damasco,61 Nállarett Dávila,62 Freddie Draper,63,40 Roosevelt García-Villacorta,64,65 Aline Lopes,21,66 Alberto Vicentini,17 Alfonso Alonso,59 Francisco Dallmeier,59 Vitor H.F. Gomes,10,67 Jon Lloyd,68 David Neill,69 Daniel Praia Portela de Aguiar,21 Luzmila Arroyo,56 Fernanda Antunes Carvalho,17,70 Fernanda Coelho de Souza,17,12 Dário Dantas do Amaral,10 Kenneth J. Feeley,71,72 Rogerio Gribel,73 Marcelo Petratti Pansonato,8,74 Jos Barlow,75 Erika Berenguer,76 Joice Ferreira,49 Paul V.A. Fine,61 Marcelino Carneiro Guedes,77 Eliana M. Jimenez,78 Juan Carlos Licona,34 Maria Cristina Peñuela Mora,79 Boris Villa,21 Carlos Cerón,80 Paul Maas,81 Marcos Silveira,82 Juliana Stropp,83 Raquel Thomas-Caesar,84 Tim R. Baker,12 Doug Daly,85 Kyle G. Dexter,86,65 Isau Huamantupa-Chuquimaco,26 William Milliken,87 Toby Pennington,35,65 Marcos Ríos Paredes,88 Alfredo Fuentes,89,90 Bente Klitgaard,91 José Luis Marcelo Pena,92 Carlos A. Peres,93 Miles R. Silman,94 J. Sebastián Tello,90 Jerome Chave,95 Fernando Cornejo Valverde,96 Anthony Di Fiore,97 Renato Richard Hilário,60 Juan Fernando Phillips,98 Gonzalo Rivas-Torres,99,100 Tinde R. van Andel,1 Patricio von Hildebrand,101 Janaína Costa Noronha,102 Edelcilio Marques Barbosa,8 Flávia Rodrigues Barbosa,102 Luiz Carlos de Matos Bonates,8 Rainiellen de Sá Carpanedo,102 Hilda Paulette Dávila Doza,88 Émile Fonty,103,7 Ricardo Zárate Gómez,104 Therany Gonzales,105 George Pepe Gallardo Gonzales,88 Bruce Hoffman,106 André Braga Junqueira,107 Yadvinder Malhi,108 Ires Paula de Andrade Miranda,8 Linder Felipe Mozombite Pinto,88 Adriana Prieto,109 Domingos de Jesus Rodrigues,102 Agustín Rudas,109 Ademir R. Ruschel,49 Natalino Silva,110 César I.A. Vela,111 Vincent Antoine Vos,112 Egleé L. Zent,113 Stanford Zent,113 Bianca Weiss Albuquerque,21 Angela Cano,52,114 Yrma Andreina Carrero Márquez,57 Diego F. Correa,52,115 Janaina Barbosa Pedrosa Costa,77 Bernardo Monteiro Flores,116 David Galbraith,12 Milena Holmgren,117 Michelle Kalamandeen,12 Marcelo Trindade Nascimento,118 Alexandre A. Oliveira,74 Hirma Ramirez-Angulo,119 Maira Rocha,21 Veridiana Vizoni Scudeller,120 Rodrigo Sierra,121 Milton Tirado,121 Maria Natalia Umaña Medina,52,122 Geertje van der Heijden,123 Emilio Vilanova Torre,119,124 Corine Vriesendorp,22 Ophelia Wang,125 Kenneth R. Young,126 Manuel Augusto Ahuite Reategui,127 Cláudia Baider,128,74 Henrik Balslev,129 Sasha Cárdenas,52 Luisa Fernanda Casas,52 William Farfan-Rios,94 Cid Ferreira,8 Reynaldo Linares-Palomino,59 Casimiro Mendoza,130,131 Italo Mesones,61 Armando Torres-Lezama,119 Ligia Estela Urrego Giraldo,36 Daniel Villarroel,56 Roderick Zagt,132 Miguel N. Alexiades,133 Edmar Almeida de Oliveira,4 Karina Garcia-Cabrera,94 Lionel Hernandez,42 Walter Palacios Cuenca,134 Susamar Pansini,30 Daniela Pauletto,135

Freddy Ramirez Arevalo,136 Adeilza Felipe Sampaio,30 Elvis H. Valderrama Sandoval,137,136 Luis Valenzuela Gamarra,23 Aurora Levesley,12 Georgia Pickavance,12 Karina Melgaço,12

8Coordenação de Biodiversidade, Instituto Nacional de Pesquisas da Amazônia - INPA, Av. André Araújo, 2936, Petrópolis, Manaus, AM, 69067-375, Brazil

9Programa Professor Visitante Nacional Sênior na Amazônia - CAPES, Universidade Federal Rural da Amazônia, Av. Perimetral, s/n, Belém, PA, Brazil

10Coordenação de Botânica, Museu Paraense Emílio Goeldi, Av. Magalhães Barata 376, C.P. 399, Belém, PA, 66040-170, Brazil

11EMBRAPA – Centro de Pesquisa Agroflorestal de Roraima, BR 174, km 8 – Distrito Industrial, Boa Vista, RR, 69301-970, Brazil

12School of Geography, University of Leeds, Woodhouse Lane, Leeds, LS2 9JT, UK

13Grupo de Investigación en Biodiversidad, Medio Ambiente y Salud-BIOMAS, Universidad de las Américas, Campus Queri, Quito, Ecuador

14Keller Science Action Center, The Field Museum, 1400 S. Lake Shore Drive, Chicago, IL, 60605-2496, USA

15Departamento de Botânica, Instituto de Pesquisas Científicas e Tecnológicas do Amapá - IEPA, Rodovia JK, Km 10, Campus do IEPA da Fazendinha, Amapá, 68901-025, Brazil

16Herbario Amazónico Colombiano, Instituto SINCHI, Calle 20 No 5-44, Bogotá, DC, Colombia

17Coordenação de Pesquisas em Ecologia, Instituto Nacional de Pesquisas da Amazônia - INPA, Av. André Araújo, 2936, Petrópolis, Manaus, AM, 69067-375, Brazil

18Dep. of Wetland Ecology, Institute of Geography and Geoecology, Karlsruhe Institute of Technology - KIT, Josefstr.1, Rastatt, D-76437, Germany

19Biogeochemistry, Max Planck Institute for Chemistry, Hahn-Meitner Weg 1, Mainz, 55128, Germany

20Naturalis Biodiversity Center, PO Box 9517, Leiden, 2300 RA, The Netherlands

21Coordenação de Dinâmica Ambiental, Instituto Nacional de Pesquisas da Amazônia - INPA, Av. André Araújo, 2936, Petrópolis, Manaus, AM, 69067-375, Brazil

22Science and Education, The Field Museum, 1400 S. Lake Shore Drive, Chicago, IL, 60605-2496, USA

23Jardín Botánico de Missouri, Oxapampa, Pasco, Peru

24Departamento de Ecologia, Universidade Estadual Paulista - UNESP – Instituto de Biociências – IB, Av. 24 A, 1515, Bela Vista, Rio Claro, SP, 13506-900, Brazil

25Divisao de Sensoriamento Remoto – DSR, Instituto Nacional de Pesquisas Espaciais – INPE, Av. dos Astronautas, 1758, Jardim da Granja, São José dos Campos, SP, 12227-010, Brazil

26Herbario Vargas, Universidad Nacional de San Antonio Abad del Cusco, Avenida de la Cultura, Nro 733, Cusco, Cuzco, Peru

27Departamento de Geografia, Universidade Estadual Paulista -UNESP – Instituto de Geociências e Ciências Extas – IGCE, Bela Vista, Rio Claro, SP, 13506-900, Brazil

28Centro de Biociências, Departamento de Ecologia, Universidade Federal do Rio Grande do Norte, Av. Senador Salgado Filho, 3000 , Natal, RN, 59072-970, Brazil

29Departamento de Biologia, Universidade Federal de Rondônia, Rodovia BR 364 s/n Km 9,5 - Sentido Acre, Unir, Porto Velho, RO, 76.824-027, Brazil

30Programa de Pós- Graduação em Biodiversidade e Biotecnologia PPG- Bionorte, Universidade Federal de Rondônia, Campus Porto Velho Km 9,5 bairro Rural, Porto Velho, RO, 76.824-027, Brazil

31Department of Biology and Florida Museum of Natural History, University of Florida, Gainesville, FL, 32611, USA

32Centre for Tropical Environmental and Sustainability Science and College of Science and Engineering, James Cook University, Cairns, Queensland, 4870, Australia

33Instituto de Investigaciones de la Amazonía Peruana (IIAP), Av. A. Quiñones km 2,5, Iquitos, Loreto, 784, Peru

34Instituto Boliviano de Investigacion Forestal, Av. 6 de agosto #28, Km. 14, Doble via La Guardia, Casilla 6204, Santa Cruz, Santa Cruz, Bolivia

35Geography, College of Life and Environmental Sciences, University of Exeter, Rennes Drive, Exeter, EX4 4RJ, UK

36Departamento de Ciencias Forestales, Universidad Nacional de Colombia, Calle 64 x Cra 65, Medellín, Antioquia, 1027, Colombia

37Agteca-Amazonica, Santa Cruz, Bolivia

38Facultad de Ciencias Agrícolas, Universidad Autónoma Gabriel René Moreno, Santa Cruz, Santa Cruz, Bolivia

39Natural History Museum, University of Oslo, Postboks 1172, Oslo, 318, Norway

40International Center for Tropical Botany (ICTB) Department of Biological Sciences, Florida International University, 11200 SW 8th Street, OE 243, Miami, FL, 33199, USA

41Cirad UMR Ecofog, AgrosParisTech,CNRS,INRA,Univ Guyane, Campus agronomique, Kourou Cedex, 97379, France

42Centro de Investigaciones Ecológicas de Guayana, Universidad Nacional Experimental de Guayana, Calle Chile, urbaniz Chilemex, Puerto Ordaz, Bolivar, Venezuela

43Prédio da Botânica e Ecologia, Embrapa Recursos Genéticos e Biotecnologia, Parque Estação Biológica, Av. W5 Norte, Brasilia, DF, 70770-917, Brazil

44Projeto Dinâmica Biológica de Fragmentos Florestais, Instituto Nacional de Pesquisas da Amazônia - INPA, Av. André Araújo, 2936, Petrópolis, Manaus, AM, 69067-375, Brazil

45Laboratório de Ecologia de Doenças Transmissíveis da Amazônia (EDTA), Instituto Leônidas e Maria Deane, Fiocruz, Rua Terezina, 476, Adrianópolis, Manaus, AM, 69060-001, Brazil

46Programa de Pós-graduação em Biodiversidade e Saúde, Instituto Oswaldo Cruz - IOC/FIOCRUZ, Pav. Arthur Neiva – Térreo, Av. Brasil, 4365 – Manguinhos, Rio de Janeiro, RJ, 21040-360, Brazil

47Instituto de Ciências Biológicas, Universidade Federal do Pará, Av. Augusto Corrêa 01, Belém, PA, 66075-110, Brazil

48Programa de Pós-Graduação em Ecologia, Universidade Federal do Pará, Av. Augusto Corrêa 01, Belém, PA, 66075-110, Brazil

49Embrapa Amazônia Oriental, Trav. Dr. Enéas Pinheiro s/nº, Belém, PA, 66095-100, Brazil

50Diretoria Técnico-Científica, Instituto de Desenvolvimento Sustentável Mamirauá, Estrada do Bexiga, 2584, Tefé, AM, 69470-000, Brazil

51Programa de Ciencias del Agro y el Mar, Herbario Universitario (PORT), UNELLEZ-Guanare, Guanare, Portuguesa, 3350, Venezuela

52Laboratorio de Ecología de Bosques Tropicales y Primatología, Universidad de los Andes, Carrera 1 # 18a- 10, Bogotá, DC, 111711, Colombia

53Programa de Pós-Graduação em Biologia (Botânica), Instituto Nacional de Pesquisas da Amazônia - INPA, Av. André Araújo, 2936, Petrópolis, Manaus, AM, 69067-375, Brazil

54Institute of Biodiversity and Ecosystem Dynamics, University of Amsterdam, Sciencepark 904, Amsterdam, 1098 XH, The Netherlands

55Endangered Species Coalition, 8530 Geren Rd., Silver Spring, MD, 20901, USA

56Museo de Historia Natural Noel Kempff Mercado, Universidad Autónoma Gabriel Rene Moreno, Avenida Irala 565 Casilla Post al 2489, Santa Cruz, Santa Cruz, Bolivia

57Facultad de Ciencias Forestales y Ambientales, Instituto de Investigaciones para el Desarrollo Forestal, Universidad de los Andes, Via Chorros de Milla, 5101, Mérida, Mérida, Venezuela

58Inventory and Monitoring Program, National Park Service, 120 Chatham Lane, Fredericksburg, VA, 22405, USA

59Center for Conservation and Sustainability, Smithsonian Conservation Biology Institute, 1100 Jefferson Dr. SW, Suite 3123, Washington, DC, 20560-0705, USA

60Universidade Federal do Amapá, Ciências Ambientais, Rod. Juscelino Kubitschek km2, Macapá, AP, 68902-280, Brazil

61Department of Integrative Biology, University of California, Berkeley, CA, 94720-3140, USA

62Biologia Vegetal, Universidade Estadual de Campinas, Caixa Postal 6109, Campinas, SP, 13.083-970, Brazil

63Department of Global Ecology, Carnegie Institution for Science, 260 Panama St., Stanford, CA, 94305, USA

64Institute of Molecular Plant Sciences, University of Edinburgh, Mayfield Rd, Edinburgh, EH3 5LR, UK

65Tropical Diversity Section, Royal Botanic Garden Edinburgh, 20a Inverleith Row, Edinburgh, Scotland, EH3 5LR, UK

66Department of Ecology, University of Brasilia, Brasilia, DF, 70904-970, Brazil

67Programa de Pós-Graduação em Ciência Ambientais, Universidade Federal do Pará, Rua Augusto Corrêa 01, Belém, PA, 66075-110, Brazil

68Faculty of Natural Sciences, Department of Life Sciences, Imperial College London, Silwood Park, South Kensington Campus, London, SW7 2AZ, UK

69Ecosistemas, Biodiversidad y Conservación de Especies, Universidad Estatal Amazónica, Km. 2 1/2 vía a Tena (Paso Lateral), Puyo, Pastaza, Ecuador

70Universidade Federal de Minas Gerais, Instituto de Ciências Biológicas, Departamento de Genética, Ecologia e Evolução, Av. Antônio Carlos, 6627 Pampulha, Belo Horizonte, MG, 31270-901, Brazil

71Department of Biology, University of Miami, Coral Gables, FL, 33146, USA

72Fairchild Tropical Botanic Garden, Coral Gables, FL, 33156, USA

73Diretoria de Pesquisas Científicas, Instituto de Pesquisas Jardim Botânico do Rio de Janeiro, Rio de Janeiro, RJ, Brazil

74Instituto de Biociências - Dept. Ecologia, Universidade de Sao Paulo - USP, Rua do Matão, Trav. 14, no. 321, Cidade Universitária, São Paulo, SP, 05508-090, Brazil

75Lancaster Environment Centre, Lancaster University, Lancaster, Lancashire, LA1 4YQ, UK

76Environmental Change Institute, University of Oxford, Oxford, Oxfordshire, OX1 3QY, UK

77Empresa Brasileira de Pesquisa Agropecuária, Embrapa Amapá, Rod. Juscelino Kubitschek km 5, Macapá, Amapá, 68903-419, Brazil

78Grupo de Investigación en Tecnologías de la Información y Medio Ambiente, Instituto Tecnológico de Antioquia - Institución Universitaria, Calle 78B No. 72A-220, Medellín, Colombia

79Universidad Regional Amazónica IKIAM, Km 7 via Muyuna, Tena, Napo, Ecuador

80Escuela de Biología Herbario Alfredo Paredes, Universidad Central, Ap. Postal 17.01.2177, Quito, Pichincha, Ecuador

81Taxonomy and Systematics, Naturalis Biodiversity Center, PO Box 9517, Leiden, 2300 RA, The Netherlands

82Museu Universitário / Centro de Ciências Biológicas e da Natureza / Laboratório de Botânica e Ecologia Vegetal, Universidade Federal do Acre, Rio Branco, AC, 69915-559, Brazil

83Institute of Biological and Health Sciences, Federal University of Alagoas, Av. Lourival Melo Mota, s/n, Tabuleiro do Martins, Maceio, AL, 57072-970, Brazil

84Iwokrama International Programme for Rainforest Conservation, Georgetown, Guyana

85New York Botanical Garden, 2900 Southern Blvd, Bronx, New York, NY, 10458-5126, USA

86School of Geosciences, University of Edinburgh, 201 Crew Building, King's Buildings, Edinburgh, EH9 3JN, UK

87Natural Capital and Plant Health, Royal Botanic Gardens, Kew, Richmond, Surrey, TW9 3AB, UK

88Servicios de Biodiversidad EIRL, Jr. Independencia 405, Iquitos, Loreto, 784, Peru

89Herbario Nacional de Bolivia, Universitario UMSA, Casilla 10077 Correo Central, La Paz, La Paz, Bolivia

90Center for Conservation and Sustainable Development, Missouri Botanical Garden, P.O. Box 299, St. Louis, MO, 63166-0299, USA

91Department for Identification & Naming, Royal Botanic Gardens, Kew, Richmond, Surrey, TW9 3AB, UK

92Department of Forestry Management, Universidad Nacional Agraria La Molina, Avenido La Molina, Apdo. 456, La Molina, Lima, Peru

93School of Environmental Sciences, University of East Anglia, Norwich, NR4 7TJ, UK

94Biology Department and Center for Energy, Environment and Sustainability, Wake Forest University, 1834 Wake Forest Rd, Winston Salem, NC, 27106, USA

95Laboratoire Evolution et Diversité Biologique, CNRS and Université Paul Sabatier, UMR 5174 EDB, Toulouse, 31000, France

96Andes to Amazon Biodiversity Program, Madre de Dios, Madre de Dios, Peru

97Department of Anthropology, University of Texas at Austin, SAC 5.150, 2201 Speedway Stop C3200, Austin, TX, 78712, USA

98Fundación Puerto Rastrojo, Cra 10 No. 24-76 Oficina 1201, Bogotá, DC, Colombia

99Colegio de Ciencias Biológicas y Ambientales-COCIBA & Galapagos Institute for the Arts and Sciences-GAIAS, Universidad San Francisco de Quito-USFQ, Quito, Pichincha, Ecuador

100Department of Wildlife Ecology and Conservation, University of Florida, 110 Newins-Ziegler Hall, Gainesville, FL, 32611, USA

101Fundación Estación de Biología, Cra 10 No. 24-76 Oficina 1201, Bogotá, DC, Colombia

102ICNHS, Federal University of Mato Grosso, Av. Alexandre Ferronato 1200, Setor Industrial, Sinop, MT, 78.557-267, Brazil

103Direction régionale de la Guyane, ONF, Cayenne, F-97300, French Guiana

104PROTERRA, Instituto de Investigaciones de la Amazonía Peruana (IIAP), Av. A. Quiñones km 2,5, Iquitos, Loreto, 784, Peru

105ACEER Foundation, Jirón Cusco N° 370, Puerto Maldonado, Madre de Dios, Peru

106Amazon Conservation Team, Doekhieweg Oost #24, Paramaribo, Suriname

107Institut de Ciència i Tecnologia Ambientals, Universitat Autònoma de Barcelona, 08193 Bellaterra, Barcelona, Spain

108Environmental Change Institute, Oxford University Centre for the Environment, Dyson Perrins Building, South Parks Road, Oxford, England, OX1 3QY, UK

109Instituto de Ciencias Naturales, Universidad Nacional de Colombia, Apartado 7945, Bogotá, DC, Colombia

110Instituto de Ciência Agrárias, Universidade Federal Rural da Amazônia, Av. Presidente Tancredo Neves 2501, Belém, PA, 66.077-830, Brazil

111Escuela Profesional de Ingeniería Forestal, Universidad Nacional de San Antonio Abad del Cusco, Jirón San Martín 451, Puerto Maldonado, Madre de Dios, Peru

112Universidad Autónoma del Beni José Ballivián, Campus Universitario Final, Av. Ejercito, Riberalta, Beni, Bolivia

113Laboratory of Human Ecology, Instituto Venezolano de Investigaciones Científicas - IVIC, Ado 20632, Caracas, Caracas, 1020A, Venezuela

114Cambridge University Botanic Garden, 1 Brookside., Cambridge, CB2 1JE, UK

115School of Agriculture and Food Sciences - ARC Centre of Excellence for Environmental Decisions CEED, The University of Queensland, St. Lucia, QLD 4072, Australia

116University of Campinas, Plant Biology Department, Rua Monteiro Lobato, 255, Cidade Universitária Zeferino Vaz, Barão Geraldo, Campinas, São Paulo, CEP 13083-862, Brazil

117Resource Ecology Group, Wageningen University & Research, Droevendaalsesteeg 3a, Lumen, building number 100, Wageningen, Gelderland, 6708 PB, The Netherlands

118Laboratório de Ciências Ambientais, Universidade Estadual do Norte Fluminense, Av. Alberto Lamego 2000, Campos dos Goyatacazes, RJ, 28013-620, Brazil

119Instituto de Investigaciones para el Desarrollo Forestal (INDEFOR), Universidad de los Andes, Conjunto Forestal, 5101, Mérida, Mérida, Venezuela

120Departamento de Biologia, Universidade Federal do Amazonas - UFAM – Instituto de Ciências Biológicas – ICB1, Av General Rodrigo Octavio 6200, Manaus, AM, 69080-900, Brazil

121GeoIS, El Día 369 y El Telégrafo, 3° Piso, Quito, Pichincha, Ecuador

122Department of Biology, University of Maryland, College Park, MD, 20742, USA

123University of Nottingham, University Park, Nottingham, NG7 2RD, UK

124School of Environmental and Forest Sciences, University of Washington, Seattle, WA, 98195-2100, USA

125Environmental Science and Policy, Northern Arizona University, Flagstaff, AZ, 86011, USA

126Geography and the Environment, University of Texas at Austin, 305 E. 23rd Street, CLA building, Austin, TX, 78712, USA

127Medio Ambiente, PLUSPRETOL, Iquitos, Loreto, Peru

128The Mauritius Herbarium, Agricultural Services, Ministry of Agro-Industry and Food Security, Reduit, 80835, Mauritius

129Department of Bioscience, Aarhus University, Building 1540 Ny Munkegade, Aarhus C, Aarhus, DK-8000, Denmark

130FOMABO, Manejo Forestal en las Tierras Tropicales de Bolivia, Sacta, Cochabamba, Bolivia

131Escuela de Ciencias Forestales (ESFOR), Universidad Mayor de San Simon (UMSS), Sacta, Cochabamba, Bolivia

132Tropenbos International, Lawickse Allee 11 PO Box 232, Wageningen, 6700 AE, The Netherlands

133School of Anthropology and Conservation, University of Kent, Marlowe Building, Canterbury, Kent, CT2 7NR, UK

134Herbario Nacional del Ecuador, Universidad Técnica del Norte, Quito, Pichincha, Ecuador

135Instituto de Biodiversidade e Floresta, Universidade Federal do Oeste do Pará, Rua Vera Paz, Campus Tapajós, Santarém, PA, 68015-110, Brazil

136Facultad de Biologia, Universidad Nacional de la Amazonia Peruana, Pevas 5ta cdra, Iquitos, Loreto, Peru

137Department of Biology, University of Missouri, St. Louis, MO, 63121, USA
